# Electrochemical Detection of Heavy Metals Using Graphene-Based Sensors: Advances, Meta-Analysis, Toxicity, and Sustainable Development Challenges

**DOI:** 10.3390/bios15080505

**Published:** 2025-08-04

**Authors:** Muhammad Saqib, Anna N. Solomonenko, Nirmal K. Hazra, Shojaa A. Aljasar, Elena I. Korotkova, Elena V. Dorozhko, Mrinal Vashisth, Pradip K. Kar

**Affiliations:** 1 Chemical Engineering Division, School of Earth Sciences and Engineering, National Research Tomsk Polytechnic University, 30 Lenin Avenue, 634050 Tomsk, Russia; ans51@tpu.ru (A.N.S.); eikor@tpu.ru (E.I.K.); elena-dorozhko@yandex.ru (E.V.D.); 2 UNESCO Laboratory of Environmental Electrochemistry, Department of Analytical Chemistry, Faculty of Science, Charles University, Hlavova 8/2030, 128 43 Prague 2, Czech Republic; 3 Department of Chemistry, Coastal Environmental Studies Research Centre, Egra Sarada Shashi Bhusan College, Vidyasagar University, Egra 721429, West Bengal, India; nirmalkrhazra@gmail.com; 4 National Research Tomsk State University, 36 Lenin Avenue, 634045 Tomsk, Russia; shujaaljasar93@gmail.com (S.A.A.); mrinalmanu10@gmail.com (M.V.); 5 Parasitology Laboratory, Department of Zoology, Cooch Behar Panchanan Barma University, Vivekananda Street, Cooch Behar 736101, West Bengal, India; karpradip@gmail.com

**Keywords:** electrochemical sensor, graphene derivatives, heavy metals, meta-analysis, food product

## Abstract

Contamination of food with heavy metals is an important factor leading to serious health concerns. Rapid identification of these heavy metals is of utmost priority. There are several methods to identify traces of heavy metals in food. Conventional methods for the detection of heavy metal residues have their limitations in terms of cost, analysis time, and complexity. In the last decade, voltammetric analysis has emerged as the most prominent electrochemical determination method for heavy metals. Voltammetry is a reliable, cost-effective, and rapid determination method. This review provides a detailed primer on recent advances in the development and application of graphene-based electrochemical sensors for heavy metal monitoring over the last decade. We critically examine aspects of graphene modification (fabrication process, stability, cost, reproducibility) and analytical properties (sensitivity, selectivity, rapid detection, lower detection, and matrix effects) of these sensors. Furthermore, to our knowledge, meta-analyses were performed for the first time for all investigated parameters, categorized based on graphene materials and heavy metal types. We also examined the pass–fail criteria according to the WHO drinking water guidelines. In addition, the effects of heavy metal toxicity on human health and the environment are discussed. Finally, the contribution of heavy metal contamination to the seventeen Sustainable Development Goals (SDGs) stated by the United Nations in 2015 is discussed in detail. The results confirm the significant impact of heavy metal contamination across twelve SDGs. This review critically examines the existing knowledge in this field and highlights significant research gaps and future opportunities. It is intended as a resource for researchers working on graphene-based electrochemical sensors for the detection of heavy metals in food safety, with the ultimate goal of improving consumer health protection.

## 1. Introduction

Contamination with heavy metals (HMs) is a serious concern due to their toxic properties and lethal effects. Heavy metals contaminate water, air, and soil and are a major contributing factor to modern environmental pollution. These heavy metals enter the environment either from natural or anthropogenic sources. Major anthropogenic sources include industrial effluents, construction and demolition waste, agricultural chemicals (pesticides, fertilizers), mining activities, medicine, and cosmetic industries. Natural sources include soil erosion, volcanic activity, forest fires, weathering of rocks and minerals, biological processes, and decomposition of organic matter. From these sources, heavy metal particles are released into the air, water, and soil. Heavy metals are not biodegradable and accumulate in the environment and in the tissues of flora and fauna [1]. Chronic exposure or acute high-dose exposure to heavy metals causes mortality in flora and fauna.

Heavy metals disrupt aquatic ecosystems and terrestrial plants. The accumulation of heavy metals in plants disrupts the normal chlorophyll synthesis process [2]. The detrimental effects of heavy metal contamination extend to aquatic flora and fauna. For instance, heavy metal contaminants accumulating in fish gills cause asphyxiation, blood poisoning, and ultimately, death [3]. Airborne heavy metals are inhaled and accumulate in tissues [4]. It is estimated that more than 13% of the world’s arable land and almost 40% of surface water sources (lakes and rivers) are contaminated with heavy metals [5].

The prevalent heavy metals or metalloids that pose a major risk to human health are chromium, mercury, lead, cadmium, and arsenic [6]. These five metals are also termed toxic to humans, but other metals, such as copper and iron, can become toxic if the required limits are exceeded. The US Food and Drug Administration (FDA) and other international authorities have also expressed concern regarding heavy metal toxicity in food and water. International organizations such as the United Nations (UN), United Nations Environment Program (UNEP), European Observatory for Nanomaterials (EUON), and government organizations of countries are working on the prevention and regulation of heavy metal intoxication [7]. Apart from contaminated raw materials, the use of preservatives and food additives is a major source of heavy metal contamination of food. The FDA has clear guidelines that prohibit the use of certain heavy metals in food and set strict limits on heavy metals in certain food additives [8]. The FDA issues updated guidelines and recommendations every year. The UN adopted the Aarhus Protocol on Heavy Metals in 1998 [9].

Voltammetric techniques offer remarkable advantages over high-performance liquid chromatography (HPLC) and spectrophotometry in the detection of heavy metals. These techniques offer increased sensitivity in the trace ranges (parts per billion), enabling analysis in intricate matrices like environmental water without requiring complex pre-concentration processes. These techniques also enable simultaneous detection of multiple metals, unlike many atomic absorption/emission spectrophotometric approaches, which are generally focused on one element at a time. Furthermore, voltammetry utilizes less expensive, portable instrumentation compared to advanced and stationary systems required for HPLC or inductively coupled plasma (ICP) spectrophotometry, allowing rapid in situ evaluation [10,11].

Graphene derivatives, such as graphene (GR), graphene oxide (GO), and reduced graphene oxide (rGO), exhibit improved electrochemical properties compared to traditional carbon materials [12,13]. This improvement is primarily due to their remarkably high specific surface area, which increases the number of active sites available for reactions, and an extended conjugated structure that promotes rapid electron transfer [14]. The distinct two-dimensional (2D) architecture of these materials contributes to better conductivity, particularly in rGO [15]. In addition, the abundant oxygen-containing functional groups in GO facilitate simple chemical modifications and improve interactions with analytes [16]. As a result, these properties lead to higher sensitivity, lower detection thresholds, and faster response times in electrochemical sensing applications. Furthermore, laser reduced graphene oxide (LRGO) sensors have enhanced the electroanalytical response for antibodies [17], biological compounds [18], and viruses [19] due to the high surface conductivity.

**Figure 1:**
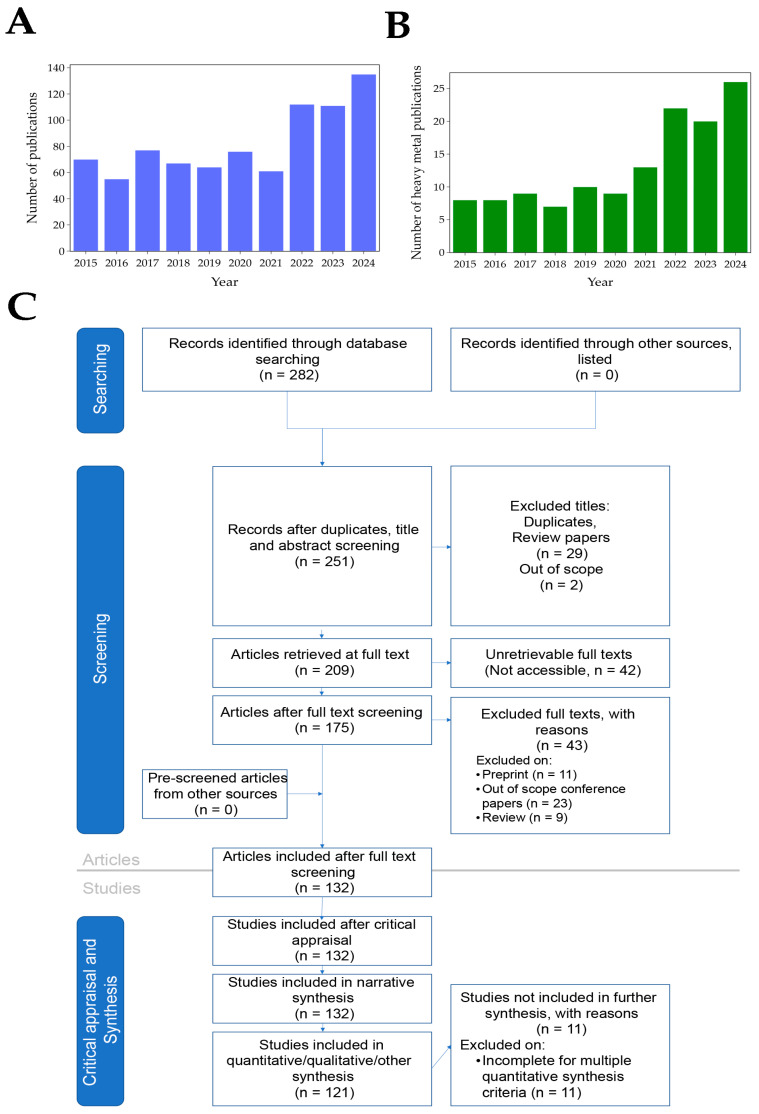
Number of publications between 2015–2024: (**A**) Development of electrochemical sensors based on graphene materials. (**B**) Development of sensors based on graphene derivatives for heavy metal detection. (**C**) ROSES flow diagram for the publications identified for meta-analysis.

In this review, the Scopus database was searched yearly (2014–2024) for publications containing the following keywords: electrochemical sensor, electrochemical biosensors, graphene, graphene oxide, and reduced graphene oxide (Figure 1A). Figure 1B shows the number of publications related to graphene-based sensors and biosensors for the electrochemical determination of heavy metals. Additionally, publications specifically on heavy metals were searched with the following keywords: electrochemical sensor, electrochemical biosensor, heavy metals, heavy metal, heavy metal ions, graphene, graphene oxide, and reduced graphene oxide. Analysis of the search results (Figure 1B) indicates that the number of publications on graphene-based electrochemical sensors for the detection of heavy metals between 2020 and 2024 increased by over 114% compared to the previous five-year period. The publications meeting search and screening criteria are organized according to the RepOrting standards for Systematic Evidence Syntheses (ROSES) [20]. The ROSES flow diagram is shown in Figure 1C.

This review is organized as follows: Section 2 focuses on critical reviews electrochemical applications of graphene-based sensors and biosensors for detection of heavy metals, Section 3 focuses on critical reviews of electrochemical applications of graphene oxide-based sensors for the detection of heavy metals, and Section 4 focuses on critical reviews of electrochemical applications of reduced graphene oxide-based sensors and biosensors for the detection of heavy metals. Section 5 provides, to the best of our knowledge, the first meta-analysis of the sensors and biosensors reviewed in Section 2, Section 3 and Section 4, focusing on their key performance metrics (LOD, LDR), comparative analytical performance grouped by heavy metal ion, and advantages/disadvantages (summarized via a coding scheme). Additionally, Section 6 provides a brief overview of the toxic effects of heavy metals on human health, plants, soil ecosystems, and aquatic life. Finally, Section 7 discusses, for the first time, the role of heavy metals in the context of the UN Sustainable Development Goals.

## 2. Electrochemical Applications of Graphene-Based Sensors and Biosensors for the Detection of Heavy Metals

Graphene-based electrochemical sensors and biosensors for the quantitative determination of heavy metals are broadly organized based on the sensor modifier type: (1) metal or metal oxide; (2) polymer; (3) other functional materials; (4) ionic liquid. Subsequently, we analyze each group in detail, highlighting the advantages and disadvantages of existing sensors and biosensors (Table 1). This conditional subdivision facilitates reading; however, it is necessary to understand that all sensing elements are not used as mono-modifiers. Sensor modifiers are often combined with GR of varying composition. Furthermore, the certain oxygen-containing groups can be combined with GR to create functional materials that facilitate the binding of heavy metal ions.

### 2.1. Metal or Metal Oxide Nanoparticle-Modified Graphene Sensors and Biosensors

#### 2.1.1. Metal Nanoparticle-Modified Graphene Sensors and Biosensors

Nanoparticles (NPs) of metals or their oxides show high catalytic activity. Graphene modified with such NPs is used for the detection of heavy metal ions due to its high chemical stability, large surface area, and ease of fabrication [21,22,23,24]. Due to the emerging synergistic interactions, this type of sensor has been widely adapted [25]. Thus, in 2010, Gong et al. used graphene decorated with gold nanoparticles (AuNPs) for the detection of Hg^2+^ [26]. The detection limit was 6 ppt, which is lower than the World Health Organization (WHO) guideline. In 2014, Zhu et al. synthesized a gold nanoparticle–graphene–cysteine composite (AuNPs/GR/L-cys) with a modified bismuth film electrode and used it for the simultaneous determination of Cd^2+^ and Pb^2+^ by square wave anodic stripping voltammetry (SWASV) [27]. The function of AuNPs is to increase the electrode conductivity and facilitate electron transfer due to quantum size effects. They proposed that the integrated properties of AuNPs, graphene, and cysteine form a nanohybrid material, enhancing the sensitivity of metal deposition to electrodes through synergistic effects. They also noted that cysteine acts as a selective metal-chelating ligand, and its incorporation into the AuNPs/GR composite significantly improves electrode modification. Despite the excellent analytical performance of these sensors, their main disadvantages are high cost and limited long-term stability.

**Figure 2:**
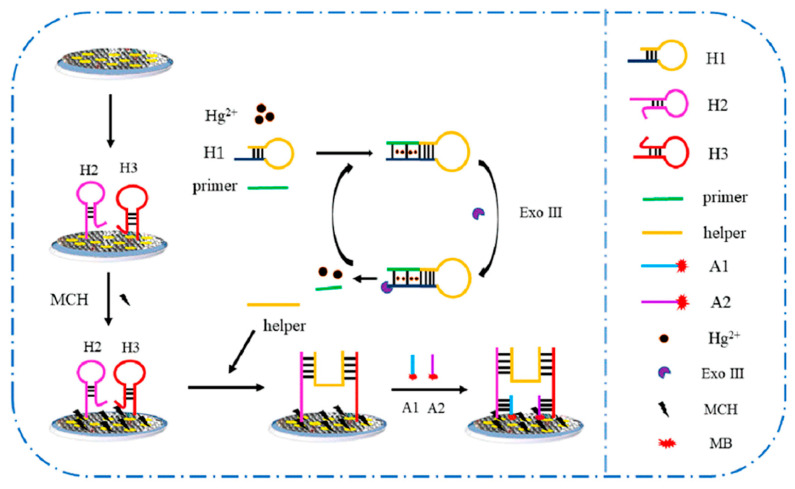
Schematic protocol of the electrochemical Hg^2+^ aptasensor (Reproduced with permission from [29], Copyright 2023 MDPI).

Graphene aerogel (GA) is often used for electrochemical heavy-metal sensors due to its porous 3D network [28], which offers a large surface area and rapid electron transport [29]. An aptasensor for the detection of Hg^2+^ ions in milk using a composite of graphene aerogel and an amplified Au nanoparticles (GAs-AuNps) composite on an indium tin oxide (ITO) substrate was fabricated by Peng et al. [29] (Figure 2). The functional mechanism of the sensor is based on a triple-amplification strategy, in which GAs-AuNPs amplify DNA loading and electron transfer, followed by exonuclease III (Exo III) mediated cyclic amplification of helper DNA strands, and finally, an H-shaped DNA structure amplifies the methylene blue (MB) detection signal. The continuous sp^2^ carbon scaffold and uniformly dispersed AuNPs synergistically lower charge-transfer resistance (Rct decreases from 156 Ω to 17 Ω), enabling femtomolar detection of Hg^2+^ (LOD = 0.16 fM) in milk samples. Thiolated DNA strands (H_2_ and H_3_) bind covalently to AuNPs via Au–S bonds, and thus, provide a dense and well-orientated layer of aptamers for efficient detection of Hg^2+^ ions. However, the detection time is hampered by a long incubation time of 4 h despite the short deposition time (225 s). The complex and costly preparation steps for GAs-AuNPs limit their rapid deployment in the field.

Nanoporous carbon (NPC) has great potential for the detection of heavy metals in real objects. Cui [30] fabricated NPC decorated with bismuth nanoparticles (Bi NPs) on graphene sheets via pyrolytic deposition. The resulting composite exhibits a large surface area and pore size, as well as good conductivity. Under optimized conditions, the BiNPs@NPCGS-based sensor was able to simultaneously determine Pb^2+^ and Cd^2+^ with detection limits of 3.2 and 4.1 nM, respectively, using SWASV. The in situ Bi-film is not a continuous film but an ultrafine layer of 2 nm Bi NPs uniformly anchored in the 3.4 nm pores of the NPCGS made of N-doped nanoporous carbon on graphene, acting as a catalytic micro-alloy that causes quantitative pre-concentration of the heavy metals at −1.0 V. The large surface area scaffold accelerates electron transfer and suppresses hydrogen evolution, enabling rapid reoxidation of the deposited metals. Bismuth nanoparticles/films are widely used [31,32,33,34] as they improve analytical characteristics through the formation of a “fused alloy” with the target metal ions. The disadvantages of such modified electrodes are a narrow potential window (below the oxidation potential of Bi) and the easy oxidation of Bi in contact with air [35], which is difficult in mass production. A disposable sensor was fabricated [36] for simultaneous detection of Cd^2+^ and Pb^2+^ ions in soil and water samples for in situ heavy metal monitoring. For this purpose, a CO_2_ laser-induced porous graphene (LIPG)on a polyimide substrate was modified using Nafion and a bismuth layer (Bi/Nafion/LIPG). The electrochemical properties of the analyte were investigated with SWASV at an accumulation time of 180 s. The thin (2 μm), highly hydrated 1 wt % Nafion layer allows Cd^2+^ and Pb^2+^ to diffuse through it in <1 s. Consequently, the 180 s pre-concentration step remains the rate-limiting factor, and the apparent response time of the electrode is unchanged compared to the uncoated LIPG. The Nafion coating acts as a cation exchange membrane, excluding interfering substances, stabilizing the Bi-layer, and enabling good recovery rates for Cd^2+^ and Pb^2+^ detection. The sensor showed an ultra-low detection limit and a wide linear range. The sensor was stable for 21 days, and the fabrication took 2–3 s due to laser induction. However, a ferrocyanide masking agent was used to mask interference from Cu^2+^. The analytical scope and added complexity due to the pre-treatment steps are the limitations of this sensor [36].

#### 2.1.2. Metal Oxide Nanoparticle-Modified Graphene Sensors

Metal oxide nanoparticles are used less frequently than metal nanoparticles [31,37,38], potentially due to the stability of such electrodes. Yukird J. et. al. [38] developed ZnO nanorods to construct a ZnO/GR nanocomposite on a screen-printed electrode (SPCE) modifier to determine Pb^2+^ and Cd^2+^ in wastewater using SWASV. The ZnO nanorods act as spacers between graphene sheets and form a hierarchical 3D structure that prevents graphene agglomeration. This configuration increases the electroactive surface area (2600 m^2^ g^−1^ vs. 640 m^2^ g^−1^ for pure graphene) and provides ample adsorption sites for heavy metal ions. Hydroxyl groups (–OH) on the surface of ZnO nanorods facilitate coordination with Cd^2+^ and Pb^2+^ via Lewis acid–base interactions and thus enable selective pre-concentration of the target ions on the electrode surface during the deposition step. Bi^3+^ increases sensitivity by forming a fused Bi-Cd/Pb alloy during the −1.2 V deposition step, which promotes co-reduction of the target metal and produces sharp, resolved anodic stripping voltammetry (ASV) peaks. Unfortunately, the article lacks information on the long-term stability of ZnO/GR/SPCE. Overall, metal oxide graphene nanocomposites enable heavy metal detection with low detection limits and good recovery in complex samples [39,40].

Hao et al. [39] developed a nanocomposite of Co_3_O_4_ nanowires and N-doped graphene aerogel (Co_3_O_4_@NGA-180) on a glassy carbon electrode for Pb^2+^ and Cd^2+^ in pond water samples. The sensor achieved a low detection limit of 2.90 μg·L^−1^ for Pb^2+^, 2.14 μg·L^−1^ for Cd^2+^, and a moderately wide LDR of 20.7–414 μg·L^−1^ for Pb^2+^ and 11.2–225 μg·L^−1^ for Cd^2+^ due to the synergistic effect of Co_3_O_4_@NGA-180, which enhances the electron transfer and ion adsorption. The 3D macroporous, nitrogen-doped graphene aerogel (NGA) provides an ultra-high surface area (117.6 m^2^ g^−1^) and interconnected channels that facilitate the deep diffusion of heavy metal ions (HMIs) and maximize adsorption sites. Nitrogen doping increases the affinity of HMIs through electrostatic interactions and coordination with pyridinic/pyrrolic N-sites. Co_3_O_4_ nanowires (diameter: ~18 nm) embedded in NGA films act as catalytic centres. During the pre-concentration step at −1.4 V, Co^2+^ oxidises to Co^3+^ and simultaneously promotes the reduction of adsorbed Pb^2+^/Cd^2+^ to Pb^0^/Cd^0^. In the stripping step, the reoxidation of the metal atoms to ions is accelerated by the simultaneous reduction of Co^3+^ to Co^2+^, which increases the anodic current. The narrow LDR and long differential pulse anodic stripping voltammetry (DPASV) accumulation time of 360 s limits the practicality for higher concentration samples and rapid field testing. Furthermore, the fabrication time includes a hydrothermal, dialysis, and freeze-drying step of 52 h to avoid calcination. This makes the sensor fabrication complicated and further limits rapid fabrication [39].

### 2.2. Polymer-Modified Graphene-Based Sensors

Various conducting polymers, including polyamidoamine, polyaniline, polydopamine, and poly(3,4-ethylenedioxythiophene), are commonly used as electrode modifiers because their numerous reactive centers enhance sensitivity for heavy metal detection [35,41]. Ruecha N. et al. [42] developed an SPCE-based electrode incorporating GR and polyaniline for the quantitative determination of Zn^2+^, Cd^2+^, and Pb^2+^ in human serum using SWASV. In this work, two methods were used, both drop-casting and a custom-built electrospray system to apply the modifier. The electrospray deposition of the modifier demonstrated higher conductivity and a larger surface area, which significantly improved the sensitivity. Nafion was additionally used to pre-concentrate metal ions. However, the reproducibility and stability of these electrodes are low, which may be due to the hydrolysis of the auxiliary electrode.

A modifier combining polyaniline, GR, and polystyrene was used [43]. Eletrospinning technology was employed to fabricate the sensor for the simultaneous determination of Pb^2+^ and Cd^2+^ in river water. In combination with GR, such fibers have a number of advantages, such as a large specific surface area, high porosity, and high mechanical properties. However, this study did not report reproducibility and stability, and the modifier production time was lengthy.

A one-dimensional graphene nanoribbon with polypyrrole modification (1D GNR-PPy) on a GCE substrate was prepared by Kulkarni et al. [44], mainly for the detection of Pb^2+^ in lake water samples using differential pulse voltammetry (DPV). The GNR-PPy composite provides a large surface area and efficient electron transfer pathways, resulting in high sensitivity and a low detection limit. The π-π interactions and hydrogen bonding between GNR and PPy further enhance the stability and performance of the composite. The Pb^2+^ ions in the solution are adsorbed on the surface of the GNR-PPy composite. The functional groups on the edges of the GNRs and the nitrogen atoms in PPy form complexes with Pb^2+^ ions, facilitating adsorption. However, the study lacks an optimization of accumulation time and an in-depth investigation of the other heavy metals. For a broader applicability of the sensor in the environment, validation for complex matrices is also required.

### 2.3. Sensors Based on Other Functional Material-Modified Graphene

#### 2.3.1. MOF-Modified Graphene-Based Sensors and Biosensors

Metal–organic frameworks (MOFs) [45], metal sulphides [46], nanoporous carbon [30], and covalent organic frameworks (COFs) [47] are also used in conjunction with graphene as modifiers of various electrodes for the quantitative simultaneous determination of different heavy metals. MOFs are widely used in sensor materials due to their unique properties such as high specific surface area, tunable pore size, customizable structure, and chemical resistance. Despite all these advantages, MOFs are often used in conjunction with carbon substrates (especially graphene and its derivatives) due to their low conductivity and mechanical stability. Graphene aerogel, characterized by high porosity, is typically synthesized by exposing graphene oxide to high temperatures followed by freeze drying [48].

In another work by Huo et al. [49], a three-dimensional graphene-based amino-functionalized metal–organic framework sensor on SPCE (3DGO/UiO-66-NH_2_ MOF/SPCE) was prepared for the electrochemical detection of Cd^2+^, Pb^2+^, Cu^2+^, and Hg^2+^ in rice, milk, and honey matrices. The surface NH_2_ groups of UiO-66-NH_2_ act as Lewis bases that rapidly chelate Cd^2+^, Pb^2+^, Cu^2+^, and Hg^2+^. The microporous architecture of the MOF (50–60 nm octahedron) and the 3D graphene network increase the effective surface area and ensure high localized enrichment. The synergy between the MOF’s large surface area (for ion uptake) and 3DGO’s conductivity provides femtomolar detection limits and good selectivity in complex food matrices. The sensor has a low deposition time of 150 s in DPV and 94–107% recovery rates [49]. However, the sensor exhibits a narrow LDR, and its stability remains untested beyond 12 days, and reliance on spiked samples may limit practical application. Another sensor for Pb^2+^ and Cd^2+^ simultaneously in water was produced by Li et al. [50]. In this sensor, an NH_2_-UiO-66 MOF grafted with ethylenediaminetetraacetic acid (EDTA) and a graphene composite side (EDTA-NH_2_-UiO-66/G) was used. The large specific surface area of MOF (893.7 m^2^ g^−1^) and the high conductivity of graphene synergistically enhance the effective electroactive sites and signal transduction, resulting in wide linear ranges and excellent anti-interference performance. EDTA, covalently grafted onto NH_2_-UiO-66, forms stable EDTA complexes with Pb^2+^ and Cd^2+^ via its abundant carboxyl groups, efficiently pre-concentrating the target ions. Under optimized conditions, Pb^2+^ is reduced to Pb^0^ on the composite surface. Subsequent anodic stripping reoxidizes Pb^0^ to Pb^2+^, producing a sharp stripping peak whose current is proportional to metal concentration, thereby enabling a low detection limit. Although the sensor is selective to the common interferents Cu^2+^ and Fe^3+^, a longer accumulation time of 510 s by DPASV prolongs the analyses. The sensor is stable over 20 days and shows promising potential after optimizing for the accumulation time.

#### 2.3.2. COF-Modified Graphene Sensors

In addition to MOFs, COFs have recently emerged as key materials for the fabrication of modified electrodes [51]. COFs are a new type of crystalline porous polymers containing light elements (C, H, O, N, B) whose building blocks and chemical bonds provide excellent sites for heavy metal ions binding via strong coordination. At the same time, they have very low conductivity. Such materials are, therefore, used in conjunction with metal nanoparticles, graphene, or in combination with these.

For example, Yu L. et al. [47] developed a new sensor based on AuNPs/GR/COF_DPTB_ for simultaneous detection of Cd^2+^, Pb^2+^, and Cu^2+^. COF_DPTB_ is a yellow powder and was synthesized using the aldehyde–amine Schiff-base condensation reaction of 1,3,5-tri(4-aminophenyl) benzene (TAPB) and 2,5-dimethoxy-*p*-phenyldiformaldehyde (DMTP) in two days. AuNPs/GR/COF_DPTB_/GCE was prepared by the drop method, in which AuNPs/GR/COF_DPTB_ was drop-casted onto the GCE surface, dried under an infrared lamp, and finally coated with Nafion. The synergistic effect of COF_DPTB_ with AuNPs and GR improves adsorption capacity and increases conductivity and electron transfer efficiency, resulting in enhanced sensor performance. The sensor achieved low LODs (Cd^2+^—3.025 nM, Pb^2+^—5.849 nM, Cu^2+^—9.993 nM) and high reproducibility. However, the properties of the electrode require further improvement in terms of stability, affordability, and fabrication. In another work, Yu et al. [52] fabricated a GCE-based sensor functionalized with GR/COF_DPTB_ for the simultaneous detection of Cd^2+^, Pb^2+^, and Cu^2+^ in baijiu. The incorporation of GR increases the electrical conductivity and provides a larger surface area that facilitates efficient electron transfer during the redox reactions. The synergistic effect of COF_DPTB_ and GR leads to improved sensitivity and selectivity. The sensor can simultaneously detect Cd^2+^, Pb^2+^, and Cu^2+^ as the peak-to-peak distances in the stripping voltammograms differ significantly, indicating that the ions do not interfere with each other. This is attributed to the selective coordination ability of the N and O atoms on COFDPTB with the metal ions. During the pre-concentration step, the adsorbed metal ions (Cd^2+^, Pb^2+^, Cu^2+^) are reduced to their metallic form at a deposition potential of −1.2 V for 260 s. The sensor thus demonstrates multiplex real-time analyses of heavy metal ions in a complex matrix.

#### 2.3.3. Other Functional Material-Modified Graphene Sensors and Biosensors

Functionalization represents another approach for utilizing GR in heavy metal ion sensors. The doping of graphene with chemical additives leads to an increase in electrocatalytic activity and sensitivity. Nitrogen-doped GR (N-GR) demonstrated this behavior [53], where it was, for the first time (deposited on GCE), as a working electrode for simultaneous determination of Hg^2+^, Cu^2+^, Cd^2+^, and Pb^2+^ in tap water. N-doped GR is a promising material as it does not require long fabrication, is stable for at least 3 weeks, is cheap, and has a wide linear range for the analyzed heavy metals. Unfortunately, LOD and selectivity are not competitive with existing sensors. Sensors incorporating various forms of graphene have been developed for the quantitative determination of heavy metal ions. Pure GR is less commonly used than graphene oxide (GO) or reduced graphene oxide (rGO), potentially due to its hydrophobicity or properties leading to less stable sensors [14].

**Figure 3:**
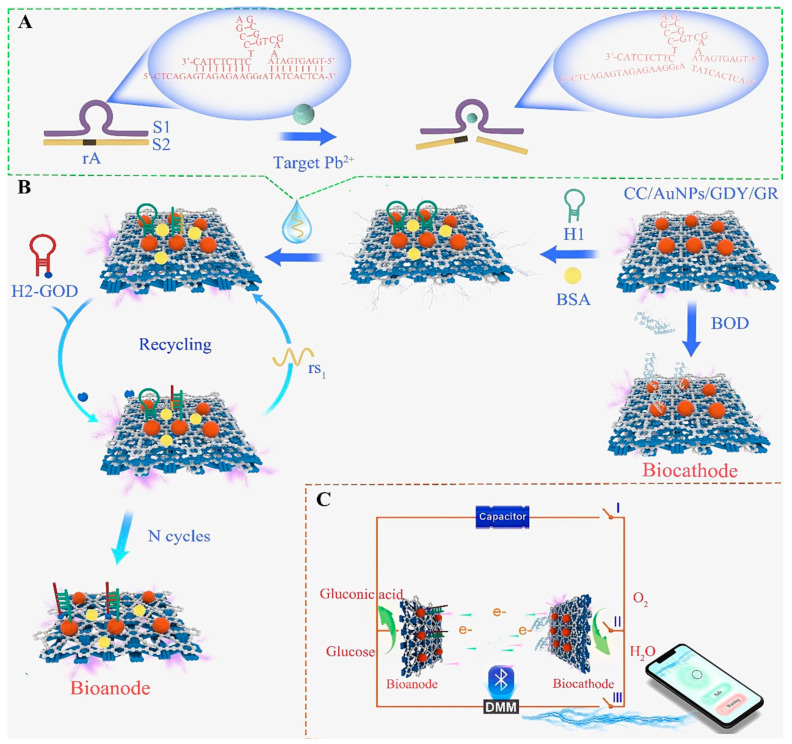
Construction of sensing platform. Pb^2+^ acts on specific sites (rA) of DNAzyme (**A**). Specific assembly process of the bioelectrode (**B**). Structure of EBFC and capacitor integrated device (**C**) (Reproduced with permission from Ref. [54], Copyright 2024 Elsevier B.V.).

Another study [54] developed a self-powered graphdiyne/graphene (GDY/GR) heterojunction with DNAzyme-triggered catalytic hairpin assembly (CHA) decorated with AuNPs on a carbon cloth electrode substrate (Figure 3). When Pb^2+^ ions are present in the sample, they interact with the DNAzyme and cause a conformational change that leads to cleavage of the DNAzyme at a specific site. This cleavage produces a smaller DNA fragment (rS1), which then initiates the CHA reaction. The CHA process amplifies the signal by forming several DNA duplexes labelled with glucose oxidase, which, in turn, generate a measurable electrochemical signal through glucose oxidation. The biosensor showed an ultrasensitive LOD of 1 nM (0.001 µg·L^−1^) for Pb^2+^ and a wide LDR of 0.003–5000 nM (0.0006–1035 µg·L^−1^). The GDY/GR heterojunction enhances electron transfer and catalytic activity, while the DNAzyme increases target binding specificity. However, the fabrication time and the process are complicated due to the long preparation time in several steps. Finally, the GDY/GR synthesis using van der Waals epitaxy may limit the scalability of the sensor. Furthermore, the platform is limited to Pb^2+^ detection [54], restricting its broader applicability. Key limitations include the lack of multiplexing capability and the complex, multi-step preparation process.

Sarà et al. developed a modified screen-printed carbon electrode (SPCE) using graphene quantum dots and tetraphenylporphyrin sulfonate (TPPS) for electrochemical detection of Cd^2+^ and Cu^2+^ in seawater, employing square wave voltammetry with an accumulation time of 180 s. The heavy metal ions (Cd^2+^ and Cu^2+^) adsorb on the surface of the GQDs@TPPS modified electrode. The TPPS component in the supramolecular system forms coordination complexes with these metal ions, enhancing their interaction with the electrode surface. However, limitations include a narrow linear dynamic range due to dual ranges, cross-interference between Cd^2+^ and Cu^2+^, and the long deposition time. Although the sensor showed moderate stability over 100 days, the requirement for acidic conditions (pH 4) and the inability to detect simultaneously hinder its applicability in the environment [55]. Laser-reduced graphene and graphene sheets-based sensors are also promising for heavy metal ions detection but face limitations, including slow detection and stability issues in food safety applications [56,57].

### 2.4. Ionic Liquid-Modified Graphene Sensors

The synergistic effect arising from the combination of ionic liquids (ILs) and GR as electrode modifiers, characterized by high ionic conductivity, wide electrochemical windows, and enhanced conductivity, underpins the promise of ILs in electrochemistry. ILs are compounds that consist entirely of ions and have a melting point below 100 °C. In this context, two papers are presented, in which GR and IL have been used as sensor modifiers for the detection of heavy metals since 2015. In [58], Bagheri H. et al. proposed an electrochemical sensor based on GR, modified with 1-n-octylpyridine hexafluorophosphate (as an IL) and a new synthetic phosphorus ylide [2,4-Cl_2_C_6_H_3_C(O)CHPPh_3_]. The working mechanism of this sensor relies on the synergistic effects of graphene, ionic liquid, and phosphorus ylide to enhance the adsorption and electrochemical detection of heavy metal ions. The graphene provides a conductive support with a large surface area, the ionic liquid improves dispersion and conductivity, and the phosphorus ylide provides selective binding sites for the metal ions. This combination results in a highly sensitive and selective sensor that can detect traces of Tl^+^, Pb^2+^, and Hg^2+^ in complex matrices. However, the preparation of such a modifier takes at least two days and requires a considerable number of reagents.

In contrast, sensor preparation in [32] is less time-consuming, but reproducibility and stability are compromised for the quantitative determination of Zn^2+^, Cd^2+^, and Pb^2+^ in drinking water by the SWASV. The working mechanism of this sensor is based on the synergistic effects of graphene, ionic liquid, and Nafion to enhance the adsorption and electrochemical detection of heavy metal ions. The graphene provides a conductive support with a large surface area, the ionic liquid improves dispersion and conductivity, and the Nafion offers selective binding sites and ion transport properties. The metal ions are pre-concentrated on the electrode surface by applying a negative potential (−1.4 V) for a certain time (120 s). This step ensures that the metal ions are reduced and deposited on the electrode surface. These enhanced analytical properties result from improved conductivity, mass transfer, and increased surface area imparted by the GR, IL, and Nafion modifier.

**Table 1:** Graphene-based electrochemical sensors and biosensors for heavy metal detection in real objects (food, water, human serum).

Electrode Substrate	Sensing Materials	Heavy Metal	Method (Accumulation Time)	(Simultaneous) LDR,µg·L^−1^	(Simultaneous) LOD, µg·L^−1^	Analytical Characteristics		Sensor Characteristics		Matrix	Reference
						Adv.	Disadv.	Adv.	Disadv.		
CC	EBFC/GDY/GR/AuNPs/DNAzyme/CHA	Pb^2+^	EIS (90 min)	0.0006–1035	0.001	A, B, C, D, F	E	G, H	–	Orange juice, tap water	[54]
CFs	Ag/GDs/NCs/CFs	^a^ Cd^2+^ ^b^ Pb^2+^	DPV (420 s)	^a^ 1405–28,102 ^b^ 2590–51,800	^a^ 2.8 ^b^ 5.2	A, B, C, D, F	E	H, I	G, J	River water	[59]
CPE	IL/GR/L	^b^ Pb^2+^ ^d^ Hg^2+^ ^e^ Tl^2+^	SWASV (90 s)	^b,d,e^ 0.3–41	^b^ 0.09 ^d^ 0.08 ^e^ 0.07	A, B, C, D, E, F	–	H, J	G, I	Tap and river water, soil	[58]
GCE	L-cys/GR/CS	^a^ Cd^2+^ ^b^ Pb^2+^	DPASV (120 s)	^a^ 0.6–67 ^b^ 1–62	^a^ 0.5 ^b^ 0.1	A, B, C, D, E, F	–	G, I	H, J	Honey, rice	[21]
	N-doped GR	^a^ Cd^2+^ ^b^ Pb^2+^ ^c^ Cu^2+^ ^d^ Hg^2+^	DPSV (300 s)	^a^ 11–101 ^b^ 2–1863 ^c^ 0.6–320 ^d^ 40–1809	^a^ 6 ^b^ 1 ^c^ 0.3 ^d^ 10	B, D, F	A, C, E	G, H, I, J	–	Tap water	[22]
	GRA/UiO-66-NH_2_ (MOF)	^a^ Cd^2+^ ^b^ Pb^2+^ ^c^ Cu^2+^ ^d^ Hg^2+^	DPSV (250 s)	^a^ 1–168 ^b^ 0.2–414 ^c^ 0.6–102 ^d^ 0.2–442	^a^ 1.1 ^b^ 0.21 ^c^ 0.5 ^d^ 0.1	A, B, D, F	C, E	J	G, H, I	River water, soil, spinach	[45]
	Bi/AuNPs/GR/L-cys	^a^ Cd^2+^ ^b^ Pb^2+^	SWASV (800 s)	^a,b^ 0.5–40	^a^ 0.1 ^b^ 0.05	A, B, D, F	C, E	J	G, H, I	Spring water	[27]
	BiNPs@NPGCS	^a^ Cd^2+^ ^b^ Pb^2+^	SWASV (180 s)	^a^ 9–90 ^b^ 12–124	^a^ 0.5 ^b^ 0.7	A, D, E, F	B, C	J	G, H, I	Lake and tap water	[30]
	Bi/Fe_2_O_3_/GR	^a^ Cd^2+^ ^b^ Pb^2+^ ^f^ Zn^2+^	DPASV (300 s)	^a,b,f^ 1–100	^a^ 0.08 ^b^ 0.07 ^f^ 0.1	A, D, F	B, C, E	I, J	G, H	Tap water	[31]
	PbS/GR	Cu^2+^	CV	1.1–11,200	1.1	A, B, E, F	C, D	–	G, H, I, J	–	[46]
	GR/N/Bi	^a^ Cd^2+^ ^b^ Pb^2+^ ^f^ Zn^2+^	DPASV (300 s)	^a,b,f^ 5–100	^a^ 0.07 ^b^ 0.05 ^f^ 0.57	A, D, F	B, C, E	I, J	G, H	Tap water	[34]
	MgFe-LDH/GR	^a^ Cd^2+^ ^b^ Pb^2+^	SWASV (180 s)	^a^ 11–112 ^b^ 21–207	^a^ 0.7 ^b^ 0.6	A, D, E, F	B, C	I, J	F, H	Lake and tap water	[37]
	GR/CeO_2_	^a^ Cd^2+^ ^b^ Pb^2+^ ^c^ Cu^2+^ ^d^ Hg^2+^	DPASV (120 s)	^a^ 22–280 ^b^ 41–518 ^c^ 13–160 ^d^ 40–503	^a^ 0.02 ^b^ 0.02 ^c^ 0.01 ^d^ 0.06	A, E, F	B, C, D	G, I, J	H	–	[23]
	Sn/poly(p-ABSA)/GR	Cd^2+^	SWASV (120 s)	1–70	0.05	A, B, C, D, E	–	G, H, I, J	–	Lake, farmland irrigation water	[24]
	COF/AuNPs/GR	^a^ Cd^2+^ ^b^ Pb^2+^ ^c^ Cu^2+^	DPASV (290 s)	^a^ 56–2016 ^b^ 104–1242 ^c^ 32–384	^a^ 0.3 ^b^ 1.2 ^c^ 0.6	A, C, D, F	B, E	–	G, H, I, J	Baijiu	[47]
	Co_3_O_4_@NGA-180	^a^ Cd^2+^ ^b^ Pb^2+^	DPASV (360 s)	^a^ 11.2–225 ^b^ 20.7–414	^a^ 2.14 ^b^ 2.90	A, C, D, F	E	G, H, I	–	Pond water	[39]
	NMO-GR	^b^ Pb^2+^ ^d^ Hg^2+^	SWASV (40 s)	^b^ 290–1596 ^d^ 140–1343	^b^ 10.4 ^d^ 5.4	A, B, C, D, F	–	G, H, I, J	–	River water	[40]
	ZMO-GR	^b^ Pb^2+^ ^d^ Hg^2+^	SWASV (40 s)	^b^ 290–1596 ^d^ 140–1343	^b^ 16.6 ^d^ 8.0						
	PEI/CS/GN	Pb^2+^	DPASV (360 s)	0.5–90	0.01	A, B, C, D, F	E	G, H, I	J	Tap water, river, lake	[60]
	GNR-PPy	Pb^2+^	DPV (180 s)	0.0207–207.2	0.00622	A, B, C, D, F	–	G, H, I, J	–	Lake water	[44]
	EDTA-NH_2_-UiO-66/G	^a^ Cd^2+^ ^b^ Pb^2+^	DPASV (510 s)	^a^ 28.1–4215 ^b^ 51.8–7770	^a^ 9.33 ^b^ 17.6	A, B, C, D, F	E	H, J	G	Tap water	[50]
	G/COF-SH	^a^ Cd^2+^ ^b^ Pb^2+^ ^c^ Cu^2+^ ^d^ Hg^2+^	SWV (400 s)	^a^ 1–1000 ^b^ 1–800 ^c^ 1–800 ^d^ 5–1000	^a^ 0.3 ^b^ 0.2 ^c^ 0.2 ^d^ 1.1	A, C, D, F	E	G, H, J	–	Coastal water	[51]
	GR/COFDPTB	^a^ Cd^2+ c^ Pb^2+ c^ Cu^2+^	DPASV (260 s)	^a^ 11–2810 ^b^ 21–2279 ^c^ 6–699	^a^ 1.24 ^b^ 1.81 ^c^ 0.41	A, B, C, D, F	–	G, H, I	–	Baijiu	[52]
	GrCFa	Pb^2+^	SWV (120 s)	829–3312	740	C, D, F	A, B	G, I	H, J	Lipstick	[61]
	STB/Gs-2 composite	^a^ Cd^2+^ ^b^ Pb^2+^ ^d^ Hg^2+^	SWASV (150 s)	^a^ 28.1–337.2 ^b^ 51.7–620.4 ^d^ 50.2–602.5	^a^ 0.13 ^b^ 0.08 ^d^ 0.12	A, B, C, D, E, F	–	G, I, J	H	River water, tap water	[57]
GE	AgNPs/GrNPs	^a^ Cd^2+^ ^b^ Pb^2+^ ^c^ Cu^2+^	SWASV (200 s)	^a,b,c^ 0.5–120	^a^ 0.005 ^b^ 0.001 ^c^ 0.0041	A, B, C, D, F	E	H, I	G, J	Tap water	[62]
GP	Bi-NPs@NC	^a^ Cd^2+^ ^b^ Pb^2+^ ^f^ Zn^2+^	SWASV (180 s)	^a^ 1–1400 ^b^ 1–1400 ^f^ 20–1400	^a^ 0.5 ^b^ 0.1 ^f^ 10	A, C, D, F	–	H, I	G, J	Tap water, lake water	[25]
ITO	GAs-AuNPs, DNA, Exo III, MB	Hg^2+^	DPV (225 s)	2.01 × 10^−7^–2.01	3.21 × 10^−8^	A, B, C, D, F	E	H, J	G, I	Milk	[29]
Microporous graphene foam	GF/GNFs	As^3+^	Chronoamperometry	1–50	1.0	A, C, D, F	B	I	G, H	Water	[28]
Polyimide	Bi/Nafion/LIPG	^a^ Cd^2+^ ^b^ Pb^2+^	SWASV (180 s)	^a,b^ 1–10; 10–60	^a^ 0.25 ^b^ 0.41	A, B, C, D, E, F	–	G, H, I, J	–	Soil, water	[36]
	N@Bi-LIG	^a^ Cd^2+^ ^b^ Pb^2+^	DPSV (300 s)	1.0–100.0	^a^ 0.207 ^b^ 0.410	A, B, C, D, F	E	H, I, J	G	Porcelain applique, tea	[63]
	LIGBi	^a^ Cd^2+^ ^b^ Pb^2+^	SWASV (300 s)	^a^ 2–180 ^b^ 2–180	^a^ 0.914 ^b^ 0.916	A, B, D, F	C	G, I, J	H	Tap water	[64]
	LIGBN	^a^ Cd^2+^ ^b^ Pb^2+^	SWV (1440 s)	^a^ 899–8993 ^b^ 1658–16,576	^a^ 28.1 ^b^ 43.5	B, C, E, F	–	G, I, J	–	–	[56]
SPCE	Bi film/N/IL/GR	^a^ Cd^2+^ ^b^ Pb^2+^ ^f^ Zn^2+^	SWASV (120 s)	^a,b,f^ 0.1–100	^a^ 0.06 ^b^ 0.08 ^f^ 0.09	A, C, E, F	B, D	G, I, J	H	Drinking water	[32]
	ZnO/GR	^a^ Cd^2+^ ^b^ Pb^2+^	ASV (180 s)	^a,b^ 10–200	^a^ 0.6 ^b^ 0.8	A, C, E, F	B, D	J	G, H, I	Wastewater	[38]
	N/GR/PANI	^a^ Cd^2+^ ^b^ Pb^2+^ ^f^ Zn^2+^	SWASV (240 s)	^a,b,f^ 1–300	^a^ 0.1 ^b^ 0.1 ^f^ 1.0	A, C, F	B, D, E	J	G, H, I	Human serum	[42]
	GR/PANI/PS/nanoporous fiber	^a^ Cd^2+^ ^b^ Pb^2+^	SWASV (180 s)	^a,b^ 10–500	^a^ 4.4 ^b^ 3.3	C, E, F	A, B, D	G, I	H, J	River water	[43]
	3DGO/UiO-66-NH_2_	^a^ Cd^2+^ ^b^ Pb^2+^ ^c^ Cu^2+^ ^d^ Hg^2+^	DPV (150 s)	^a^ 0.00112–0.0393 ^b^ 0.00207–0.0725 ^c^ 0.000635–0.0222 ^d^ 0.00201–0.0702	^a^ 0.00123 ^b^ 0.00124 ^c^ 0.000184 ^d^ 0.000622	A, C, D, E, F	B	G, H, I, J	–	Rice, milk, honey	[49]
	GQDs@TPPS (1:6) GQDs@TPPS (1:9)	^a^ Cd^2+^ ^c^ Cu^2+^	SWV (180 s)	^a^ 0–900; 675–1463 ^c^ 0–510; 382–828	^a^ 49.0 ^c^ 10.9	A, C, D, F	B, E	G, I, J	H	Seawater	[55]

^a^—Cd^2+^, ^b^—Pb^2+^, ^c^—Cu^2+^, ^d^—Hg^2+^, ^e^—Tl^2+^, and ^f^—Zn^2+^. **Analytical characteristics**. **Advantages (Adv.): A** low LOD; **B** wide LDR; **C** selective electrode; **D** high reproducibility; **E** low deposition time; **F** high conductivity. **Disadvantages (Disadv.): A** high LOD; **B** narrow LDR; **C** non-selective electrode; **D** low reproducibility; **E** long deposition time. **Sensor characteristics**. **Adv.: G** easy modifier fabrication; **H** long-term stability; **I** low cost; **J** fast modification of the electrode. **Disadv.: F** low conductivity; **G** complex modifier fabrication; **H** short-term stability; **I** high cost; **J** long modification of electrode.

## 3. Electrochemical Applications of Graphene Oxide-Based Sensors for the Detection of Heavy Metals

Graphene oxide (GO) is a single-layered graphite sheet containing hydroxyl, carboxyl, and epoxy oxygen groups on its basal plane and edges, resulting in a mixture of sp2 and sp3 hybridized carbon atoms [65]. GO is stable in aqueous solution due to the presence of functional groups (negative zeta potential) that cause electrostatic repulsion, stabilizing the dispersion [53]. This stability is a significant advantage for heavy metal determination in water. GO sensors are a promising way to detect heavy metals due to their unique properties and ease of use. GO, when combined with other materials, exhibits high sensitivity and selectivity towards various metals, enabling rapid and accurate detection in environmental samples (Table 2). However, the widespread use of such sensors requires further technological development and improvements of their characteristics, such as stability, manufacturing complexity, and duration of manufacturing modifiers.

The abundance of oxygen-containing groups in GO facilitates its functionalization, in contrast to other carbon nanomaterials. Functionalization of GO not only preserves its excellent properties but also adds new ones, such as high stability, dispersion, and conductivity. Currently, GO functionalization methods mainly include covalent and non-covalent functionalization and elemental doping, which is discussed in detail in the review [66]. Over the past decade, advances in this field have resulted in a variety of GO-based electrochemical sensors for the quantitative determination of heavy metals, as shown in Table 2. The most common materials used as modifiers in conjunction with GO are nanoparticles of metals or their oxides [66,67,68,69], polymers [70,71,72,73,74], MOF [75,76], carbon materials [33], and the active complex of ruthenium (II) bipyridine [77,78], the advantages and disadvantages of which we will discuss in more detail below.

### 3.1. Metal or Metal Oxide Nanoparticle-Modified Graphene Oxide Sensors

#### 3.1.1. Metal Nanoparticle-Modified Graphene Oxide Sensors

Metal nanoparticles in combination with GO are commonly used for the detection of heavy metals. Bao et al. [79] developed a portable system for detecting Hg^2+^ and Cu^2+^ in tap water using DPV. The system comprises (1) an SPCE (PET substrate) modified with chitosan/polyaniline–bismuth nanoparticles@graphene oxide and multi-walled carbon nanotubes (CS/PANi–Bi NP@GO–MWCNTs); (2) Bluetooth data transmission; and (3) a 3D-printed microelectrolytic cell. Accumulation times were 240 s for Hg^2+^ and 216 s for Cu^2+^. The functional mechanism of this sensor is based on the synergistic effects of the composite material (CS/PANi–Bi NP@GO–MWCNT) to improve the adsorption and electrochemical detection of heavy metal ions. The composite’s large surface area and functional groups facilitate the adsorption of Hg^2^ and Cu^2+^ ions, while the bismuth nanoparticles increase the electrochemical activity. The detection process begins with the adsorption of heavy metal ions (Hg^2+^ and Cu^2+^) on the modified electrode surface. The functional groups of the chitosan and the large surface area of GO and MWCNTs facilitate the adsorption of ions. The bismuth nanoparticles play a crucial role in enhancing the electrochemical activity and facilitate the detection process. The sensor fulfils the WHO/EPA safety limits. However, the narrow LDR for Cu^2+^ limits its usefulness for highly contaminated samples due to ion interference at higher concentrations. In addition, sample handling is manual, risking reproducibility. Manual sample handling risks reproducibility, limiting wider field deployment where robust operation is needed.

#### 3.1.2. Metal Oxide Nanoparticle-Modified Graphene Oxide Sensors

According to the literature review, iron oxides with GO are the most commonly used metal oxide nanoparticles with GO for the determination of heavy metals in real objects [66,68,76,79,80]. GO suspension incorporating iron oxide nanoparticles enhances electrode selectivity and adsorption capacity [81,82]. However, the agglomeration of magnetic nanoparticles hinders the analysis, so other polymers are often used as modifiers. Thus, in the work [69], ZnSe nanocomposite deposited on a copper-coated laminate was used together with GO for the voltammetric determination of cadmium in wastewater. The ZnSe/GO nanocomposite is known as a stable, reproducible, and selective material for electrode modification. Significant disadvantages include a high LOD (45.54 µM), poor selectivity, lengthy analysis, and limited stability (5 days). Improving these analytical characteristics would make the material promising for heavy metal detection. For example, [67] modified a GCE with GO decorated with manganese dioxide. Alternatively, [76] developed a GCE coated with poly(amidoamine) dendrimer-functionalized magnetic graphene oxide (GO/Fe_3_O_4_/PAMAM) for simultaneous Pb^2+^ and Cd^2+^ detection in environmental waters. The working mechanism of this sensor is based on the synergistic effects of the composite material (GO-Fe_3_O_4_-PAMAM) to improve the adsorption and electrochemical detection of heavy metal ions. The large surface area and functional groups of the composite facilitate the adsorption of Pb^2+^ and Cd^2+^ ions, while the magnetic properties of the Fe_3_O_4_ nanoparticles support their accumulation. The heavy metal ions adsorbed on the electrode surface are pre-concentrated by applying a negative potential (−1.1 V for Pb^2+^ and −1.2 V for Cd^2+^) for 160 s. The functional groups of PAMAM and the large surface area of GO provide multiple binding sites for the target ions, which increases the selectivity of the sensor. However, the fabrication time of such a sensor is about 2 days, which is a material and cost-intensive process. Other studies combine GO/Fe_3_O_4_ with metal oxide nanoparticles beyond polymers. For instance, [68] developed a SWV method using a PGE modified with GO/Fe_3_O_4_/Bi_2_O_3_ to determine Cd^2+^ in water, fruit, soil, and biological samples. The modified electrode showed better electrocatalytic activity with a higher electroactive area compared to GO/Fe_3_O_4_. The advantages of this sensor are low LOD (1.85 ng·L^−1^), long-term stability (up to 40 days), and low cost.

In addition, Ul Haq et al. [83] developed a sensor using Sb_2_WO_6_/GO nanostructures on layered GO to detect Hg^2+^ and UO_2_^2+^ in aqueous samples using DPV (240 s) with high sensitivity to Pb^2+^ and Cd^2+^ interferences. Firstly, GO has a large surface area and contains functional groups such as hydroxyl and carboxyl, and Sb_2_WO_6_ provides additional surface area and specific binding sites that can enhance the interaction with heavy metal ions. The hierarchical structure of Sb_2_WO_6_ increases the available surface area for adsorption. The oxidation peak for Hg^2+^ is observed at a potential of 0.24 V. The current of the oxidation peak increases with increasing concentration of Hg^2+^. Notably, the Sb_2_WO_6_/GO nanostructure also demonstrated photocatalytic activity, degrading tetracycline with 90% efficiency within 70 min [83]. While this dual functionality is innovative, the production time of the modifier and the complex chemical preparation steps hinder scalability beyond the laboratory setting. The narrow LDR and non-trace detection limit the use cases.

### 3.2. Polymer-Modified Graphene Oxide Sensors

Conductive polymers, possessing numerous reactive centers, effectively pre-concentrate heavy metal ions on the electrode surface, enhancing detection sensitivity. However, they also exhibit disadvantages, including low mechanical strength, poor thermal stability, and high water absorption. In this regard, the use of GO in combination with polymers is justified by the improvement of these disadvantages [84]. Moreover, not only synthetic polymers such as polyaniline [70,85], poly(3,4-ethylenedioxythiophene) [71], polypyrrole [73], and poly(L-glutamic acid) (PGA) [74] are used, but also biopolymers. Thus, Priya T. et al. used a biopolymer, κ-carrageenan (κ-Car), together with GO and the amino acid L-cysteine (L-cys), which has a strong ability to chelate metals, for the detection of Pb^2+^ and Cd^2+^ in groundwater, tap water, and raw milk using SWASV [72]. κ-carrageenan possesses numerous active hydroxyl groups and a sulphate group that facilitate metal ion binding. The κ-Car/GO/L-cys mixture was deposited by a simple “drop” method. Such a sensor has many advantages, namely selectivity, high reproducibility, and low LODs (0.58 nM for Cd^2+^ and 1.08 nM for Pb^2+^) and stability (20 days). Adsorbed heavy metal ions are pre-concentrated by applying −1.0 V for 120 s. This step ensures that the metal ions are reduced and accumulate on the electrode surface. Detection relies on the synergistic effect of complexation by SO_3_^−^, NH_2_, SH, and COO^−^ groups combined with GO’s large surface area [72]. However, the preparation of such a sensor requires the use of numerous reagents and is time-consuming. In contrast, [70] reports a shorter preparation time of 5 h for synthesizing the polyaniline hydrogel (PANI) and preparing the PANI/GO modifier. This sensor demonstrated strong performance and was applied for Pb^2+^ detection in industrial effluents and natural water. The quantitative determination of Pb^2+^ was performed using the SWASV method at a deposition time of 600 s, which showed good electroanalytical characteristics in two linear ranges of 0.2–250 nM and 250–3500 nM. The developed technique showed that the LOD is about 0.04 nM, which is significantly less than the WHO Limits.

### 3.3. MOF-Modified Graphene Oxide Sensors

MOFs are promising electrode modifiers but require combination with carbon materials due to their inherent low conductivity and stability [86]. Compared to graphene (GR), GO is often a more suitable substrate owing to its good aqueous dispersibility (from hydrophilic functional groups) and retained electrical conductivity (from sp^2^ carbon domains) [75]. This interaction was tested with zeolitic imidazolate frameworks (ZIP-8) as a MOF for the voltammetric determination of Pb^2+^ in wastewater [75]. The GCE modifier based on ZIF-8/GO was obtained by applying a ZIF-8 coating on the GO surface. This synergistic combination improves the electrochemical properties of the sensor by providing more active sites for the adsorption and detection of Pb^2+^ ions. The detection of Pb^2+^ ions is based on their redox reactions at the electrode surface. The Pb^2+^ ions are adsorbed on the ZIF-8@GO composite and undergo oxidation reactions that generate a measurable current. While relatively simple to fabricate, this modified electrode has limitations; its LOD exceeds WHO requirements for lead in water, and interference studies were incomplete.

**Figure 4:**
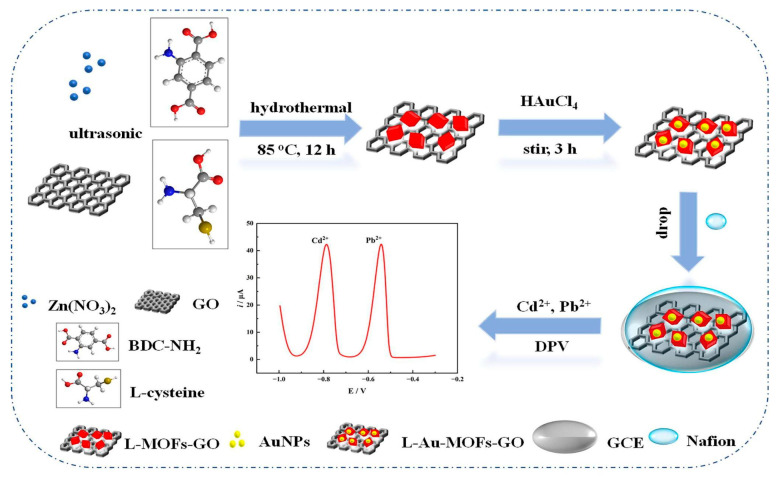
Schematic illustration of L-Au-MOFs-GO composite synthesized via the in situ growth and the detection of Cd^2+^ and Pb^2+^ by L-Au-MOFs-GO-modified electrode (Reproduced with permission from Ref. [87], Copyright 2023 Elsevier B.V.).

In another work, Wang et al. [87] developed an electrochemical sensor using L-cysteine-functionalized AuNPs/MOFs-GO nanocomposites on GCE (Nafion/L-Au-MOFs-GO/GCE) for the simultaneous detection of Cd^2+^ and Pb^2+^ in river water and watermelon samples (Figure 4). The heavy metal ions (Cd^2+^ and Pb^2+^) are adsorbed onto the composite through interactions with the hydrophilic groups of L-cysteine, MOFs, and GO. The amino nitrogen, sulfhydryl, and carboxyl oxygen groups in L-cysteine, together with the carboxyl and hydroxyl groups in GO, provide multiple binding sites for the metal ions. At an optimal pH (pH 4.5), the functional groups are less protonated, which allows for a stronger electrostatic interaction between the negatively charged functional groups and the positively charged heavy metal ions. This improves the adsorption of the target ions on the sensor surface, which leads to a higher sensitivity. However, the recovery rates vary widely (80–101%), and the LODs exceed WHO drinking water safety limits (3 µg·L^−1^ for Cd^2+^, 10 µg·L^−1^ for Pb^2+^). Additionally, the long accumulation time of 300 s in the DPV is suboptimal for real-time detection. Furthermore, the synthesis is complex and involves multi-step hydrothermal processing, which limits scalability. The sensor’s long-term stability also needs further research.

### 3.4. Non-Metallic Functional Material-Modified Graphene Oxide Sensors

#### 3.4.1. Sensors Using Active Complex of Ruthenium (II) Bipyridine-Modified Graphene Oxide

Ruthenium (II) complexes based on pyridine ligands [Ru(bpy)3]^2+^ are increasingly important for modifying electrodes to detect various analytes, including heavy metals. They enhance the mass transfer efficiency, charge transfer, sensitivity, and selectivity, and they have tunable electrochemical properties and stable oxidation states from I to III [77]. Simultaneously, GO provides high π-bonding and doping of organic or inorganic complexes, facilitating [Ru(bpy)_3_]^2+^ functionalization through electrostatic and π-π interactions. This is confirmed in [77], where a GO/[Ru(bpy)_3_]^2+^ nanocomposite deposited on an Au electrode was used for the simultaneous voltammetric determination of four heavy metals. The proposed sensor is highly sensitive to metal ions, with low LODs that meet WHO limits. In addition, GO/[Ru(bpy)_3_]^2+^/Au was shown to be easy to prepare, stable for at least 30 days, and suitable for the determination of Cd^2+^, Pb^2+^, As^3+^, and Hg^2+^ in Cauvery and tap water. In 2023, Albalawi I. et al. also confirmed the effectiveness of using GO/[Ru(bpy)_3_]^2+^ nanocomposites in the quantification of Cd^2+^ and Pb^2+^ [78]. AuNPs are used because of their excellent conductivity and stability, which can improve the electron transfer rate. The negatively charged GO and citrate-capped AuNPs interact with the positively charged [Ru(bpy)_3_]^2+^ complex, immobilizing it on the electrode surface. This interaction helps to immobilize the complex on the electrode surface. Nafion is incorporated into the composite to improve electron transfer, reduce interference, and increase long-term stability. Nafion’s cation exchange properties immobilize the [Ru(bpy)_3_]^2+^ complex on the electrode surface. Functional groups on GO and the [Ru(bpy)_3_]^2+^ complex provide binding sites for heavy metal ions (Cd^2+^ and Pb^2+^). The heavy metal ions are adsorbed on the sensor surface through electrostatic interactions with the functional groups and the immobilized [Ru(bpy)_3_]^2+^ complex. While the modifying material improves electron transfer and stability, the sensor’s selectivity is a significant disadvantage. Moreover, such a sensor can be expensive for routine analyses, despite its 30-day stability.

#### 3.4.2. Carbon Material-Modified Graphene Oxide Sensors

Carbon nanomaterials are crucial for electrochemical heavy metal sensors due to their unique physical, mechanical, and electrical properties [88]. Their use significantly increases the sensitivity, selectivity, and overall effectiveness of the sensors. In addition, the use of GO together with other carbon materials can not only fully utilize the respective advantages but also compensate for the corresponding disadvantages. Thus, a nanocomposite consisting of one-dimensional MWCNTs and two-dimensional GO films was fabricated in [33]. It was shown that the hydrophilic components of GO interact with MWCNTs, causing a certain amount of MWCNTs to “dissolve” in water, and at the same time, MWCNTs partially prevent the adhesion of GO nanosheets. This hydrophilic hybrid nanocomposite can also absorb heavy metals from water. Such a nanocomposite was deposited on GCE for the quantitative determination of Pb^2+^ and Cd^2+^ in electroplating effluent using DPASV. However, a major disadvantage is the lengthy manufacturing process for both the modifier and final sensor, hindering mass production.

**Figure 5:**
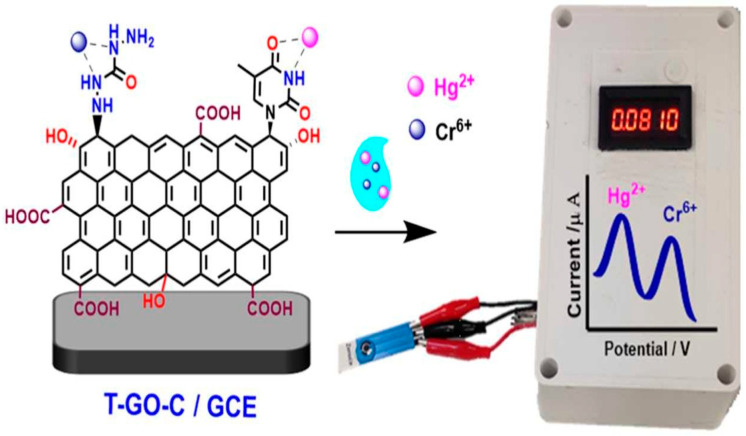
Schematic representation of the T-GO-C nanomaterial-fabricated electrochemical sensor electrode and its SWV sensing of Hg^2+^ and Cr^6+^ (Reproduced with permission from Ref. [90], Copyright 2025 Elsevier B.V.).

In 2021, Martinez Jimenez M. J. et al. [89] also used carbon nanotubes (CNTs) in combination with GO as electrode modifiers for the determination of lead in drinking water [89]. PEI-CNTs and GO are deposited on gold IDEs using the layer-by-layer technique. This method involves sequential adsorption of PEI-CNTs and GO driven by van der Waals forces, hydrogen bonding, π-π stacking, and electrostatic interactions. The layer-by-layer film formation increases the surface area and provides a patterned platform for sensing. The functional groups present in PEI-CNTs and GO have a high affinity for heavy metal ions. These functional groups can attract and bind Pb^2+^, which changes the electrical properties of the layer-by-layer film. The sensor has excellent repeatability and stability, with a standard deviation of 1.8% over 15 detection repetitions. This shows that the sensor can reliably detect traces of Pb ions in aqueous solutions. In further investigations, the resistance of the layered film (PEI-CNT/GO)25 was determined. Thymine plays a crucial role in the binding of Hg^2+^ ions due to its unique ability to form stable complexes with mercury. Jayaraman et al. [90] produced a covalently dual-functionalized graphene oxide electrochemical multiplex sensor (T-GO-C) for simultaneous detection of Hg^2+^ and Cr^6+^ in tap and tannery water samples by SWV with an accumulation time of 120 s (Figure 5). Thymine plays a crucial role in binding Hg^2+^ ions due to its ability to form stable complexes with mercury. GO is covalently functionalized with thymine and carbohydrazide (T-GO-C) by an epoxide ring-opening reaction. The sensor shows a good recovery rate and accuracy, which confirms its applicability in real-life scenarios. The sensor demonstrated excellent selectivity due to the selective binding of thymine and carbohydrazide to Hg^2+^ and Cr^6+^, respectively. Furthermore, an RSD < 4.5% indicates good reproducibility, while a moderate long-term stability of the sensor for 4 weeks and recovery rates improve the field applicability [90]. However, the high LOD for Cr^6+^ is disadvantageous compared to Hg^2+^ and is affected by competing bonds of Cd^2+^ and As^3+^. While the fabrication process is simple, it requires controlling the nitrogen atmosphere, limiting scalability.

**Table 2:** Graphene oxide-based electrochemical sensors for heavy metal detection in real objects (food, water).

Electrode Substrate	Sensing Materials	Heavy Metal	Method (Accumulation time)	(Simultaneous) LDR, µg·L^−1^	(Simultaneous) LOD, µg·L^−1^	Analytical Characteristics		Sensor Characteristics		Matrix	Reference
						Adv.	Disadv.	Adv.	Disadv.		
Au	[Ru(bpy)_3_]^2+^/GO	^a^ Cd^2+^ ^b^ Pb^2+^ ^c^ As^3+^ ^d^ Hg^2+^	DPV	^a^ 6–34 ^b^ 10–52 ^c^ 4–135 ^d^ 20–241	^a^ 0.3 ^b^ 0.3 ^c^ 0.2 ^d^ 0.3	A, D, F	B, C	H, I, J	G	Cauvery and tap river water	[77]
	PEI/CNTs/GO	Pb^2+^	Amperometry (30 s)	207–1532	16	E, F	A, B, C, D	I	G, H, J	Drinking water	[89]
Cu	ZnSe/GO	Cd^2+^	DPV (180 s)	11,200−78,400	5101	D, F	A, B, C, E	G, I	H, J	Wastewater	[69]
CPE	AC/GP (1:1)	^a^ Cd^2+^ ^b^ Pb^2+^	CV and LSV (510 s)	1–75	^a^ 27.68 ^b^ 37.78	A, C, D, F	B	G, I, J	H	Simulated wastewater	[88]
	AC/RGO (1:1)				^a^ 10.91 ^b^ 14.01	A, B, C, D, F	–	G, I, J	H		
	AC/RGO/Chitosan (1:1:1)				^a^ 33.11 ^b^ 33.70	A, C, D, F	B	G, I, J	H		
GCE	3DGO/Py10	Cd^2+^	SWASV (600 s)	5–400	3.6	D, F	A, B, C, E	H, I, J	F	Tap and lake water	[91]
	GO	^a^ Cd^2+^ ^b^ Pb^2+^	DPASV (30 min)	^a^ 0.0001–1.1 ^b^ 0.0002–2.1	^a^ 2.8 × 10^−5^ ^b^ 5.8 × 10^−4^	A, D, E, F	B, C	I, J	G, H	Rice, tap water, soya, milk	[92]
	GO/Fe_3_O_4_/2-CBT	^a^ Cd^2+^ ^b^ Pb^2+^	SWASV (180 s)	^a ,b^ 0.08–90	^a^ 0.03 ^b^ 0.02	A, B, D, E, F	C	I, J	G, H	Tap, river and wastewater	[79]
	GO/Fe3O4/ PAMAM	^a^ Cd^2+^ ^b^ Pb^2+^	SWASV (160 s)	^a^ 0.2–140 ^b^ 0.4–120	^a^ 0.07 ^b^ 0.13	A, B, C, D E, F	–	H, J	G, I	River water	[76]
	GO/Fe_3_O_4_/ PMDA/AuNPs	^c^ As^3+^ ^e^ Cu^2+^	SWASV (300 s)	^c^ 5–500 ^e^ 0.5–750	^c^ 0.2 ^e^ 0.1	A, D, F	B, C, E	J	G, H, I	Drinking and pool water	[80]
	MnFe_2_O_4_/GO	Pb^2+^	SWASV (150 s)	41–228	18	D, E, F	A, B, C	I, J	G, H	River water	[66]
	GO/MnO_2_	^a^ Cd^2+^ ^b^ Pb^2+^	DPASV (1800 s)	^a^ 0.001–11 ^b^ 0.002–21	^a^ 1.6 × 10^−5^ ^b^ 2.6 × 10^−4^	A, B, C, D, F	E	I, J	G, H	Sea water	[67]
	PANI/GO	Pb^2+^	SWASV (600 s)	0.04–52 52–725	0.008	A, B, D, F	C, E	G, H, I, J	–	Industrial effluents, natural water	[70]
	PEDOT/GO	Hg^2+^	DPSV (360 s)	2–603	0.6	A, B, D, F	C, E	H, I, J	G	Tap water	[71]
	GO/κ-Car/L-cys	^a^ Cd^2+^ ^b^ Pb^2+^	SWASV (120 s)	^a^ 0.6–6 ^b^ 1–10	^a^ 0.07 ^b^ 0.2	A, C, D, E, F	B	H, I, J	G	Ground and tap water, raw milk	[72]
	PA/PPy/GO	^a^ Cd^2+^ ^b^ Pb^2+^	DPV (200 s)	^a,b^ 5–150	^a^ 2.1 ^b^ 0.4	A, B, D, E, F	C	I, J	G, H	Tap water	[73]
	PGA/GO	^a^ Cd^2+^ ^d^ Hg^2+^ ^e^ Cu^2+^	DPASV (1600 s)	^a^ 28–616 ^d^ 50–1106 ^e^ 16–352	^a^ 1.7 ^d^ 6.4 ^e^ 1.5	A, D, F	B, C, E	H, I, J	G	Lake water	[74]
	ZIF-8/GO	Pb^2+^	DPASV (180 s)	207–41,400	223	D, E, F	A, B, C	G, I, J	H	Wastewater	[75]
	GO/MWCNTs/Bi	^a^ Cd^2+^ ^b^ Pb^2+^	DPASV (180 s)	^a,b^ 0.5–30	^a^ 0.1 ^b^ 0.2	A, B, C, E, F	D	I, J	G, H	Electroplating effluent	[33]
	T-GO-C	^d^ Hg^2+^ ^f^ Cr^6+^	SWV (120 s)	^d^ 5–600 ^f^ 5–600	^d^ 1 ^f^ 20	A, B, C, D, E, F	–	G, H, I, J	–	Tap water, tannery water	[90]
	Nafion/TEOA@AuNPs-GO-UiO-66-NH2	^a^ Cd^2+^ ^b^ Pb^2+^ ^e^ Cu^2+^	DPV (480 s)	^a,b,e^ 100–3200	^a^ 11.7 ^b^ 27.6 ^e^ 33.2	B, C, D, F	A, E	G, J	H, I	River water	[93]
	Sb_2_WO_6_/GO	^d^ Hg^2+^ ^g^ UO_2_^2+^	DPV (240 s)	^d^ 2006–24,071 ^g^ 2700–40,505	^d^ 0.762 ^g^ 37.8	A, C, D, F	–	G, I	H, J	Aqueous samples	[83]
	Nafion/L-Au-MOFs-GO	^a^ Cd^2+^ ^b^ Pb^2+^	DPV (300 s)	^a,b^ 80–560	^a^ 20.8 ^b^ 17.3	B, C, D, F	A, E	G, I, J	H	River water, watermelon	[87]
	ZIF-67/GO	^b^ Pb^2+^ ^d^ Hg^2+^ ^h^ Zn^2+^ ^i^ Cr^3+^	DPV (280 s)	^b^ 6.21–58.1 ^d^ 6–30 ^h^ 1.96–9.81 ^i^ 1.56–17.16	^b^ 0.207 ^d^ 0.12 ^h^ 0.131 ^i^ 0.26	A, C, D, F	B	G, I	J	Real water	[81]
	NF@75rGO@ABT	^a^ Cd^2+^ ^d^ Hg^2+^ ^e^ Cu^2+^	SWASV (100 s)	^a^ 0.0056–140 ^d^ 0.010–270 ^e^ 0.0032–80	^a^ 0.0138 ^d^ 0.0173 ^e^ 0.00344	A, B, C, D, E, F	–	–	G, I, J	Cosmetics, water	[82]
	GO/UiO-67@PtNPs	As^3+^	SWSV (250 s)	0.202–3.0	0.0315	A, C, D, F	B	G, J	H, I	Chinese herbal medicines	[94]
	Co_3_O_4_/GO-2/Nafion	^a^ Cd^2+^ ^b^ Pb^2+^	DPASV (120 s)	^a^ 157.4–719.4 ^b^ 62.2–1326.1	^a^ 224.8 ^b^ 9.32	A, B, C, D, E, F	–	G, H, I, J	–	Lotus pool water	[95]
PET-based SPCE	CS/PANi–Bi NP@GO–MWCNTs	^d^ Hg^2+^ ^e^ Cu^2+^	DPV (460 s)	^d^ 0–200 ^e^ 500–16,908	^d^ 10 ^e^ 998	A, C, D, F	B	G, I	–	Tap water	[96]
PGE	MGO-PANI	^a^ Cd^2+^ ^b^ Pb^2+^	DPV (384 s)	^a^ 0.5–100; 100–1000 ^b^ 0.1–50; 50–1500	^a^ 0.65 ^b^ 0.12	A, B, C, D, F	–	G, I, J	–	River water	[85]
	Bi_2_O_3_/Fe_2_O_3_/GO	Cd^2+^	SWV (90 s)	0.006–1.2	0.002	A, C, E, F	B, D	H, I, J	G	Fruits, water, soil, human serum	[68]
SPGE	[Ru(bpy)_3_]^2+^/GO/N	^a^ Cd^2+^ ^b^ Pb^2+^	SWASV (120 s)	^a,b^ 50–350	^a^ 4.2 ^b^ 5.3	A, D, E, F	B, C	H, J	G, I	River and tap water	[78]
SPCE	L-cys/GO/PPy	Pb^2+^	DPASV (600 s)	1.4–28 28–280 280–14,000	0.07	A, B, D, F	C, E	H, I, J	G	Spiked and industry water	[86]
	NH_2_-MOF@GO15%-NF carbon aerogel	^a^ Cd^2+^ ^b^ Pb^2+^	DPASV (300 s)	^a^ 1–150 ^b^ 1–150	^a^ 0.16 ^b^ 0.07	A, B, C, E, F	–	I, J	G, H	Shrimp, clam, sea snail	[97]

^a^—Cd^2+^, ^b^—Pb^2+^, ^c^—As^3+^, ^d^—Hg^2+^, ^e^—Cu^2+^, ^f^—Cr^2+^, ^g^—UO_2_^2+^, ^h^—Zn^2+^, and ^i^—Cr^3+^. **Analytical characteristics**. **Advantages (Adv.): A** low LOD; **B** wide LDR; **C** selective electrode; **D** high reproducibility; **E** low deposition time; **F** high conductivity. **Disadvantages (Disadv.): A** high LOD; **B** narrow LDR; **C** non-selective electrode; **D** low reproducibility; **E** long deposition time. **Sensor characteristics**. **Adv.**: **G** easy modifier fabrication; **H** long-term stability; **I** low cost; **J** fast modification of the electrode. **Disadv.**: **F** low conductivity; **G** complex modifier fabrication; **H** short-term stability; **I** high cost; **J** long modification of electrode.

## 4. Electrochemical Applications of Reduced Graphene Oxide-Based Sensors and Biosensors for the Detection of Heavy Metals

The oxidized functional groups in GO can be removed by reduction to produce reduced graphene oxide (rGO). The characteristics of rGO, which are significantly impacted by the reduction method used, often include considerable structural disadvantages [98]. Because rGO simplifies the fabrication of composite materials, it is often used as a modifier for an electrochemical sensor. In addition, the presence of functional groups with a negative charge in rGO enhances the preliminary concentration of metal ions (with a positive charge) around the electrode surface, which is used to adsorb metal ions [53]. Similar to GR and GO, rGO is used in electrochemical sensors alongside modifiers such as metal nanoparticles (and their oxides), polymers, and other carbon materials, and it is also integral to biosensors for heavy metal detection, justifying its classification as a separate group. The characteristics, advantages, and disadvantages of the sensors developed are listed in Table 3.

### 4.1. Metal or Metal Oxide Nanoparticle-Modified Reduced Graphene Oxide Sensors

#### 4.1.1. Metal Nanoparticle-Modified Reduced Graphene Oxide Sensors

**Figure 6:**
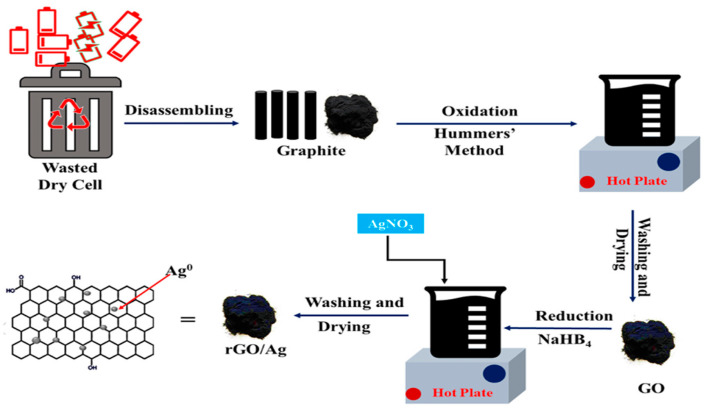
Schematic route for the preparation of Ag NPs/rGO nanocomposite (Reproduced with permission from Ref. [99], Copyright 2024 Elsevier B.V.).

Gold, silver, and bismuth nanoparticles are most commonly used in conjunction with rGO for the quantification of heavy metals. For example, in [99,100], simple modified sensors based on Au NPs [101,102], Ag NPs [99], Bi NPs [103], and rGO were developed to determine the levels of As^3+^, Cd^2+^, Pb^2+^, Cu^2+,^ and Hg^2+^, respectively. In [99], an Ag NPs/rGO nanocomposite was synthesized by chemically reducing a mixture of GO and AgNO_3_ using sodium borohydride (NaBH_4_) as both the reducing agent and stabilizer. GO is synthesized from graphite obtained from old dry cells using the modified Hummers method. This process involves the oxidation of graphite to introduce oxygen-containing functional groups that facilitate further functionalization. The synthesis process for this nanocomposite is illustrated in Figure 6.

The detection mechanism is based on the redox reactions of As^3+^ ions on the electrode surface. The reduction of As^3+^ to As^0^ and the subsequent oxidation of As^0^ back to As^3+^ generate a current signal that is measured by DPASV. The rGO component of the nanocomposite provides a large surface area and adsorption sites for As^3+^ ions, which increases the sensitivity of the sensor. The Ag NPs facilitate the electron transfer processes and thus improve the electrochemical performance. The modified Ag NPs/rGO/GCE have successfully detected As^3+^ with an LOD of 0.24 ppb and a sensitivity of 1.24 μA/ppb in water, which meets WHO requirements. This sensor demonstrates good sensitivity and selectivity, but it requires an improvement in its linear detection range. Similarly, the sensor developed in [100], which deposited Ag NPs/rGO onto a magnetic glassy carbon electrode (MGCE) for simultaneous determination of four heavy metals, also suffers from a limited linear range. Unfortunately, the use of such a sensor was not demonstrated on real objects; moreover, the LOD does not meet the WHO requirements, and there is no information regarding the reproducibility and selectivity, which is challenging for practical applications.

In addition, Au-Bi bimetallic nanoparticles combined with reduced graphene oxide (rGO/Au-Bi) were used for the simultaneous electrochemical determination of Pb^2+^ and Cd^2+^ in river water, honey, and orange juice [104]. The combination of rGO and Au-Bi bimetallic NPs enhances the sensor’s sensitivity through a synergistic effect. The large surface area of rGO increases the adsorption capacity, while the catalytic properties of Au-Bi NPs improve the electrochemical response. The heavy metal ions (Pb^2+^ and Cd^2+^) are adsorbed on the surface of the rGO/Au-Bi nanocomposites. The applied potential and the accumulation time allow the metal ions to accumulate on the electrode surface. This results in a wide LDR and low LODs of 0.05 μg·L^−1^ for Pb^2+^ and 0.02 μg·L^−1^ for Cd^2+^, along with good selectivity, although copper ions (Cu^2+^) cause interference. Adding ferrocyanide to the sample prior to measurement is suggested to mitigate Cu^2+^ interference. While relatively simple to fabricate, this sensor lacks long-term stability and would be very expensive for mass production.

#### 4.1.2. Metal Oxide Nanoparticle-Modified Reduced Graphene Oxide Sensors

Beyond metal nanoparticles, rGO has also been combined with metal oxide nanoparticles, including cobalt oxide [105], manganese oxide [106], titanium oxide [107], metal-doped oxides [108], and zinc oxides [109]. So, Luyen et al. [110] fabricated a ZnO/electrochemically reduced graphene oxide (ZnO/ErGO)-modified GCE for simultaneous detection of Cd^2+^ and Pb^2+^ via 120 s DPASV in lake, river, and tap water samples. The large electrochemically active surface area (0.130 cm^2^) and low charge transfer resistance (924 Ω) of ZnO/ErGO enable rapid and dense accumulation of metallic Pb^0^ and Cd^0^ on the electrode surface. The ZnO provides ample adsorption sites and prevents graphene restacking, while ErGO ensures fast electron transport. Furthermore, the Nafion binder, a proton-conducting perfluorinated ionomer, creates ion channels that provide additional pathways for electron hopping. The accumulation potential of –1.2 V is sufficiently negative to reduce the deposit of Pb^2+^ and Cd^2+^, enabling an ultrasensitive detection LOD of 0.45 µg·L^−1^ for Pb^2+^ and 1.69 µg·L^−1^ for Cd^2+^ with a linear dynamic range of 2.5–200 µg·L^−1^ for both ions. Furthermore, the sensor exhibits good selectivity and reproducibility (RSD ≤ 7.27%) but lower stability (5 days) with sample recoveries of 88–106.5% [110]. Furthermore, despite achieving LODs below WHO standards, the sensor’s real-world application is hindered by its comparatively narrow linear dynamic range, intermetallic interferences, and limited stability. The fabrication process is costly and involves a long modification time, limiting the scalability of the sensor. However, the sensor exhibits minimal interference, with signal changes of less than 8% for most common ions at environmentally relevant concentrations.

Similarly, the fabrication process using hydrothermal/calcination processes can hinder the scalability of the sensor despite low material cost. In another study, an rGO sensor was fabricated for the detection of heavy metals in tobacco leaves using SWASV [111]. However, the sensor requires complex modification with a multi-step fabrication process, including hydrothermal/chemical reduction, polishing, sonication, and drying, which hinders rapid field deployment. The sensor exhibits only moderate stability, retaining adequate performance for just over 15 days, which limits its suitability for long-term environmental monitoring.

### 4.2. Polymer-Modified Reduced Graphene Oxide Sensors

Combining rGO with various conductive polymers enhances dispersibility, high conductivity, and thermal stability, which is beneficial for the electrochemical determination of heavy metals [112,113,114,115,116]. In the preparation of such composites, rGO is generally used as a reinforcing element in a polymer matrix. Furthermore, rGO’s inherent hydrophobicity causes irreversible agglomeration in aqueous solutions. Incorporating polymers into rGO composites mitigates this issue by improving nanomaterial dispersion, making these composites suitable for heavy metal detection in environmental samples [117]. In 2020, an effective electrochemical sensor for real-time detection of heavy metal ions in tap water using rGO embellished with alanine and polyaniline was developed by Akhtar M. et al. [118] for the simultaneous detection of three heavy metals in tap water. The rGO serves as a highly conductive two-dimensional scaffold, offering a large surface area and enabling fast electron transfer kinetics. Furthermore, surface decoration with alanine and PANI prevents restacking of the graphene sheets. Alanine acts as a chelating ligand, while PANI enhances conductivity through its redox properties. The heavy metal ions in solution are electrochemically reduced and adsorbed on the electrode surface (−1.2 V). The advantages of such a sensor are high selectivity and reproducibility, low cost, and fast modification of the electrode. The disadvantages include the lack of information on long-term stability. The use of rGO and CS-based modifiers is also observed in [119], but with the inclusion of another polymer, polyethylenimine (PEI). In the aforementioned work [120], CS was used not only as a promising adsorbent with numerous amino and hydroxyl groups that can react with heavy metal ions but also as a reducing agent and stabilizer for the conversion of GO to rGO. In contrast, an electrochemical sensor based on rGO, chitosan (CS), and poly-L-lysine (PLL) was developed in [120] for simultaneous detection of Cd^2+^, Pb^2+^, and Cu^2+^ ions, which showed good stability. The rGO-CS/PLL/GCE provides an excellent platform for heavy metal determination due to its increased active surface area, high adsorption capacity, and the specific complexing ability of CS and PLL, as demonstrated in tap water analysis. PLL was applied to rGO-CS/GCE by electropolymerization of L-lysine using CV. The heavy metal ions are pre-concentrated on the electrode surface by applying a negative potential (–1.2 V) for an accumulation time (180 s). During this step, the metal ions are reduced in the solution and deposited on the electrode surface. The detection process is optimized by carefully controlling parameters such as the carrier electrolyte (0.1 M acetate buffer, pH 4.5), the deposition potential (–1.2 V), and the deposition time (180 s). These conditions ensure maximum adsorption and efficient stripping of the metal ions. The rGO-CS/PLL/GCE achieved low LODs of 0.01 µg·L^−1^ for Cd^2+^, 0.02 µg·L^−1^ for Pb^2+^, and 0.02 µg·L^−1^ for Cu^2+^, significantly below the WHO recommended limits for drinking water, although within a relatively LRD of 0.05–10 µg·L^−1^.

Another interesting example is the use of the oligomer calixarene (CAR) in conjunction with rGO as GCE modifiers for sub-nanomolar simultaneous determinations of Cd^2+^, Pb^2+^, and Fe^2+^ [114]. However, achieving these excellent analytical characteristics requires a long time, primarily due to the lengthy modifier preparation process [115] and long accumulation time (25 min), hindering on-site environmental monitoring.

### 4.3. Biosensors Based on Reduced Graphene Oxide

Electrochemical biosensors are widely used in electrochemistry for the detection of various analytes, including heavy metal ions, due to their high sensitivity, fast response time, and simple design [121,122]. Depending on the type of biomaterial fixed on the electrode surface, biosensors are categorized as aptasensors, cell, enzyme, and immunosensors. rGO is an excellent substrate for immobilizing biocomponents due to its high biocompatibility and chemical inertness. However, due to the van der Waals interactions in the interlayer, rGO has poor dispersion, which is why inorganic nanomaterials are added to rGO. Decorating the rGO layers with inorganic nanomaterials prevents aggregation, thereby enhancing the electrochemical properties of the resulting biosensors. In [123], an electrochemical biosensor for the detection of lead ions was developed based on the specific binding of an aptamer to Pb^2+^ and the composite CS/rGO/TiO_2_. Chitosan provides a stable and biocompatible environment for the immobilization of other materials, rGO provides a large surface area and excellent electrical conductivity, which improves electron transfer, and TiO_2_ nanoparticles are added to the composite to prevent the rGO layers from re-agglutinating, thus increasing the surface area and stability of the composite. In the presence of Pb^2+^ ions, the aptamer binds to Pb^2+^ and causes a conformational change from a double-stranded structure to a stable G-quadruplex structure. Methylene blue molecules, used as redox indicators, exhibit higher affinity for double-stranded DNA than for single-stranded DNA. When Pb^2+^ binds to the aptamer, the double-stranded DNA unwinds and releases MB from the electrode surface. The consequent release of MB from the electrode surface leads to a decrease in the electrochemical signal, measured via DPV. This signal change is proportional to the concentration of Pb^2+^ ions in the sample. The aptasensor was tested for the quantitative determination of Pb^2+^ in rice, tea, and eggs with good selectivity. However, the aptasensor’s sensitivity decreased by 75% after two weeks of storage.

**Figure 7:**
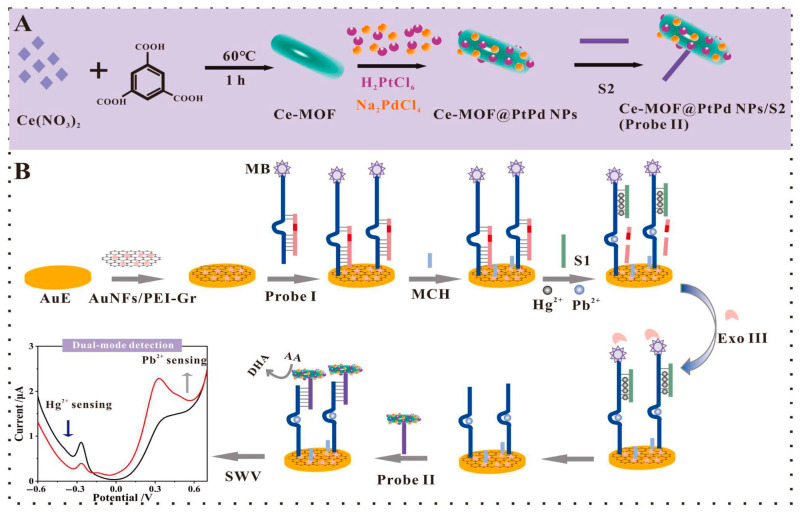
The preparation process of the signal label (Probe II) (**A**). Schematic diagram of a sensor for simultaneous detection of Hg^2+^ and Pb^2+^ (**B**) (Reproduced with permission from Ref. [124], Copyright 2024 Elsevier B.V.).

An electrochemical aptasensor [124] with a gold electrode modified with Au nanoflowers/polyethyleneimine-reduced graphene oxide (AuNFs/PEI-rGO) was fabricated to improve conductivity and surface area and to achieve high selectivity by an amplification strategy using exonuclease III (Exo III) for the simultaneous detection of Hg^2+^ and Pb^2+^ (Figure 7). The working mechanism involves Hg^2+^-induced T-Hg^2+^-T mismatches that trigger Exo III cleavage (decrease the methylene blue signal) and a Pb^2+^-activated DNAzyme cleavage that allows the Ce-MOFs@PtPd NPs to catalyze ascorbic acid (AA) oxidation (increase in AA signal). The decrease in the MB signal and the increase in the AA oxidation peak are used to quantify the concentrations of Hg^2+^ and Pb^2+^, respectively. This dual-signal approach enhances sensor selectivity. However, variable recovery rates (87–109.6%) and a long incubation time render the aptasensor unsuitable for rapid measurements, particularly in complex matrices. Furthermore, complicated fabrication steps and the requirement for expensive materials (AuNFs, Pt, and PdNPs) present significant barriers to scalability.

Another example of an rGO-based biosensor for the detection of heavy metals is the study by Yang Y. et al. [125]. The 3D-rGO/PANI nanocomposite was synthesized by chemical oxidative polymerization and then used as a sensitive DNA adsorbent layer for detecting Hg^2+^ in river water. The biosensor functions because T-rich DNA fragments bind to the amino groups of the 3D-rGO/PANI nanocomposite; these bound fragments then interact with Hg^2+^ ions to form T-Hg^2+^-T complexes. The 3D-rGO/PANI/DNA/Au electrode showed excellent analytical characteristics—a linear range from 0.1 nM to 100 nM with a low LOD of 0.035 nM—as well as high selectivity and reproducibility. However, such a DNA biosensor is expensive to fabricate and requires further studies on the stability of the biosensor. Zhang et al. [126] developed a complex sensor (HRP@ZIF-8/THI/Au/IL-rGO@GCE) for detecting heavy metals in real water samples using SWV (250 s accumulation). The detection mechanism relies on the inherent electrochemical signals of the contaminants. Signal amplification is achieved through EDTA-induced decomposition of the ZIF-8 framework, which exposes thionine (THI) and releases horseradish peroxidase (HRP). HRP then catalyzes the H_2_O_2_-mediated oxidation of THI. However, the long EDTA incubation time of 40 min is a limitation for real-time sensor applications, and the stability at room temperature limits wider application. Furthermore, while recovery rates of >88% are practical, the scalability of the sensor design is challenging, particularly due to the complex fabrication steps.

In addition, Kushwah et al. [127] produced an electrochemical aptasensor with ssDNA immobilization functionalized with AuNP-decorated reduced graphene oxide on a glassy carbon electrode (rGO/AuNPs/ssDNA@GCE) for the determination of Pb^2+^ concentration using CV at 360 s deposition time. The combination of rGO and AuNPs increases the efficiency of electron transfer. ssDNA acts as the recognition element, forming a specific bond with Pb^2+^ ions. This binding induces conformational changes detectable electrochemically. When Pb^2+^ ions are present in the sample, they bind to the ssDNA aptamer and cause a conformational change from a random coil to a G-quadruplex structure. This change affects the electron transfer properties of the electrode. The binding of Pb^2+^ to the aptamer leads to a measurable change in the electrochemical signal. The detection process is optimized by controlling parameters such as the concentration of rGO/AuNPs/ssDNA, the deposition time, the deposition potential, and the pH of the supporting electrolyte. These conditions are crucial to achieve the best electrochemical response. The sensor showed a low LOD of 1.52 nM (0.315 µg·L^−1^) over CV, which is below the EPA permissible limit (72 nM) and has good recoveries, and the LDR for this biosensor was 1.035–10.35 µg·L^−1^. The biosensor demonstrated good reproducibility (1.48% RSD) and stability, retaining 97.8% of its initial signal response after 4 weeks [127]. However, the narrow LDR limits broad environmental sensing with higher contamination in multiplex sensing requirements. In addition, a deposition time of 360 s hinders real-time sensing and a 2 h ssDNA immobilization, and the use of expensive materials such as AuNPs is a challenge for scalability.

**Table 3:** Reduced graphene oxide-based sensors and biosensors for the electrochemical detection of heavy metals in real objects (food, water, serum).

Electrode Substrate	Sensing Materials	Heavy Metal	Method (Accumulation Time)	(Simultaneous) LDR, µg·L^−1^	(Simultaneous) LOD, µg·L^−1^	Analytical Characteristics		Sensor Characteristics		Matrix	Reference
						Adv.	Disadv.	Adv.	Disadv.		
Au	3D-rGO/PANI/DNA	Hg^2+^	EIS	0.02–20	0.007	A, B, C, D, F	–	–	G, H, I, J	River water	[125]
	rGO/CNT/Bi	^a^ Cd^2+^ ^b^ Pb^2+^	SWASV (150 s)	^a,b^ 20–200	^a^ 0.6 ^b^ 0.2	A, E, F	B, C, D	J	G, H, I	Drinking water	[128]
	AuNFs/PEI-rGO	^b^ Pb^2+^ ^e^ Hg^2+^	SWV (210 min)	^b^ 0.0002–20.72 ^e^ 0.0002–20.06	^e^ 0.000022 ^b^ 0.000019	A, B, C, D, F	E	H	G, I, J	Tap water	[124]
CC	polyP NPs/rGO	^b^ Pb^2+^ ^d^ Cu^2+^	DPV (120 s)	^b^ 2.07–414 ^d^ 3.18–158.75	^b^ 0.50 ^d^ 0.07	A, C, D, E, F	–	G, H, I	–	Tap water, Lake Taiho	[112]
C-SPE	PCA-RGO/AuNPs	As^3+^	ASV (60 s)	7.5–7500	19	A, E, F	–	I, J	G	Water	[101]
GCE	CAR/rGO	^a^ Cd^2+^ ^b^ Pb^2+^ ^c^ Fe^2+^	SWV (25 min)	^a^ 0.01–1.1 ^b^ 0.02–2.1 ^c^ 0.006–0.6	^a^ 0.002 ^b^ 0.004 ^c^ 0.001	A, C, D, F	B, E	H, J	G, I	–	[114]
	T/CA/AuNPs/rGO	Hg^2+^	DPV (120 s)	0.01–1	0.002	A, C, E, F	B, D	J	G, H, I	Tap water	[129]
	ALA/pDA/rGO	^a^ Cd^2+^ ^b^ Pb^2+^ ^c^ Fe^2+^ ^d^ Cu^2+^	DPV (480 s)	^a^ 3–10 ^b^ 9–21 ^c^ 50–100 ^d^ 20–50	^a^ 1.5 ^b^ 2.9 ^c^ 50 ^d^ 18	A, D, F	B, C, E	I, J	G, H	Tap water	[130]
	rGO/PANI/HBr	^a^ Cd^2+^ ^b^ Pb^2+^	DPV (60 s)	^a^ 1–26 ^b^ 2–37	^a^ 0.7 ^b^ 1.5	A, B, C, D, E, F	–	G, I, J	H	Tap, mineral industrial wastewater, plasma	[131]
	rGO/CS/PLL	^a^ Cd^2+^ ^b^ Pb^2+^ ^d^ Cu^2+^	DPASV (180 s)	^a,b,d^ 0.05–10	^a^ 0.01 ^b^ 0.02 ^d^ 0.02	A, C, D, E, F	B	G, H	I, J	Tap water	[120]
	rGO/ALA/PANI	^a^ Cd^2+^ ^b^ Pb^2+^ ^d^ Cu^2+^	SWASV (220 s)	^a^ 0.009–11 ^b^ 0.02–21 ^d^ 0.005–6	^a^ 0.003 ^b^ 0.009 ^d^ 0.004	A, C, D, F	B, E	I, J	G, H	Tap water	[118]
	PEI/rGO/CS	Pb^2+^	DPASV (120 s)	1–130	0.005	A, B, D, E, F	C	H, I	G, J	River water	[119]
	MB/Apt/MCH/CP/CS/rGO/TiO_2_	Pb^2+^	DPV (300 s)	0.001–1	0.0003	A, C, F	B, D, E	–	G, H, I, J	Tea, rice, egg	[123]
	Au-Bi/rGO	^a^ Cd^2+^ ^b^ Pb^2+^	DPASV (200 s)	^a^ 0.1–300 ^b^ 0.1–500	^a^ 0.02 ^b^ 0.05	A, B, E, F	C, D	J	G, H, I	River water, honey, orange juice	[104]
	AuNPs/rGO	Pb^2+^	SWASV (180 s)	2–31	0.2	A, B, E, F	C, D	G, J	H, I	Tap water	[132]
	Co_3_O_4_/rGO	^a^ Cd^2+^ ^b^ Pb^2+^	SWASV (250 s)	^a,b^ 0.1–450	^a^ 0.062 ^b^ 0.034	A, B, D, F	C, E	H, I, J	G	Pool, well and tap water	[105]
	Co:ZnO/rGO	^a^ Cd^2+^ ^b^ Pb^2+^	DPV (200 s)	^a,b^ 10–90	^a^ 0.9 ^b^ 0.8	A, D, E, F	B, C	I, J	G, H	Tap water	[109]
	rGO/AgNPs	As^3+^	DPASV (120 s)	0–25	0.24	A, C, D, E, F	B	G, H, I, J	–	Water	[99]
	rGO/Zn MOF	As^3+^	DPASV (150 s)	0.2–25	0.06	A, B, C, D, E, F	–	I, J	G, H	Ground, mineral and river water	[133]
	TiO_2_/rGO	^a^ Cd^2+^ ^b^ Pb^2+^	DPASV (60 s)	^a^ 5–100 ^b^ 5–100	^a^ 3.12 ^b^ 2.38	A, C, D, E, F	–	H, I	G, J	River water	[107]
	ZnO/ErGO	^a^ Cd^2+^ ^b^ Pb^2+^	DPASV (120 s)	^a,b^ 2.5–200	^a^ 1.69 ^b^ 0.45	A, B, C, D, E, F	–	G, H, I, J	–	Tap water, river water, lake water	[110]
	NCO/N,S-rGO	^a^ Cd^2+^ ^b^ Cu^2+^ ^e^ Hg^2+^	DPASV (120 s)	^a^ 16.8–168 ^b^ 9.54–95.4 ^e^ 30.1–301	^a^ 6.63 ^b^ 4.89 ^e^ 32.8	A, C, D, E, F	B	I	H	Real water	[108]
	rGO/MoS_2_/CS	Pb^2+^	SWASV (180 s)	1.036–414.4	0.3315	A, B, C, D, E, F	–	H	G, I, J	Tobacco leaves	[111]
	MnO_2_@RGO	^a^ Cd^2+^ ^f^ Zn^2+^ ^d^ Cu^2+^	DPASV (330 s)	^a^ 0.05–700 ^f^ 0.05–600 ^d^ 0.05–600	^a^ 0.015 ^f^ 0.002 ^d^ 0.0093	A, B, C, D, F	E	G, H, I, J	–	Surface water	[106]
	Cys-CTS/N-RGO	Cu^2+^	DPASV (480 s)	15.89–8897	6.36	A, B, C, D, E, F	–	G, H, I	J	Tap water, bottled water, river water	[113]
	HRP@ZIF-8/THI/Au/IL-rGO	^b^ Pb^2+^ ^c^ Fe^2+^ ^d^ Cu^2+^ ^e^ Hg^2+^ ^f^ Zn^2+^ ^g^ Ba^2+^ ^h^ Co^2+^ ^i^ Cr^2+^ ^j^ Mn^2+^	SWV (250 s)	^b^ 0.207–2072 ^c^ 0.056–559 ^d^ 0.064–636 ^e^ 0.201–2006 ^f^ 0.065–654 ^g^ 0.137–1373 ^h^ 0.059–589 ^i^ 0.052–520 ^j^ 0.055–549	^b^ 0.0273 ^c^ 0.00680 ^d^ 0.00871 ^e^ 0.0269 ^f^ 0.0103 ^g^ 0.0155 ^h^ 0.00927 ^i^ 0.00750 ^j^ 0.00639	A, B, C, D F	–	H	G, J	Real water samples	[126]
	rGO/AuNPs/ssDNA	Pb^2+^	CV (360 s)	1.035–10.35	0.315	A, C, D, E, F	B	G, H	J	Tap, pond, river, lake water	[127]
MGCE	AgNPs/rGO	^a^ Cd^2+^ ^b^ Pb^2+^ ^d^ Cu^2+^ ^e^ Hg^2+^	SWASV (150 s)	^a^ 6–392 ^b^ 10–518 ^d^ 3–224 ^e^ 101–603	^a^ 28 ^b^ 59 ^d^ 11 ^e^ 36	E, F	A, B, C, D	G, I, J	H	–	[100]
Ni foam	Ni/TiO_2_/P-rGO/CS	Cu^2+^	DPV (200 s)	0–286	0.014	A, B, C, D, F	–	H, I	G	Seawater	[116]
Polyimide	Nafion/BiNP@LIG	^a^ Cd^2+^ ^b^ Pb^2+^	SWASV (200 s)	^a^ 5–50 ^b^ 5–50	^a^ 0.5 ^b^ 0.8	A, C, D, F	–	G, I, J	H	Soil	[103]
SPE	rGO/AuNPs	As^3+^	DPV (120 s)	100–1000	100	A, C, D, F	–	G, H	I	Human serum	[102]
	rGO/Bi film	^a^ Cd^2+^ ^b^ Pb^2+^	SWASV (150 s)	^a,b^ 1–60	^a^ 0.5 ^b^ 0.8	A, B, E, F	C, D	G, I, J	H	Milk	[134]
SPCE	TTU/rGO	Hg^2+^	DPV (600 s)	100–180,000	26	D, F	A, B, C, E	H, I, J	G	River water	[135]
	AO/rGO	^a^ Cd^2+^ ^b^ Pb^2+^	SWASV (120 s)	^a^ 56–1120 ^b^ 104–2070	^a^ 10 ^b^ 2	A, D, E, F	B, C	J	G, H, I	Human plasma	[136]
	4AP/rGO	Pb^2+^	DPV (300 s)	0.0004–0.04	0.0001	A, C, F	B, D, E	I, J	G, H	Tap water	[137]

^a^—Cd^2+^, ^b^—Pb^2+^, ^c^—Fe^2+^, ^d^—Cu^2+^, ^e^—Hg^2+^, ^f^—Zn^2+^, ^g^—Ba^2+^, ^h^—Co^2+^, ^i^—Cr^2+^, and ^j^—Mn^2+^. **Analytical characteristics. Advantages (Adv.): A** low LOD; **B** wide LDR; **C** selective electrode; **D** high reproducibility; **E** low deposition time; **F** high conductivity. **Disadvantages (Disadv.): A** high LOD; **B** narrow LDR; **C** non-selective electrode; **D** low reproducibility; **E** long deposition time. **Sensor characteristics**. **Adv.**: **G** easy modifier fabrication; **H** long-term stability; **I** low cost; **J** fast modification of the electrode. **Disadv.**: **G** complex modifier fabrication; **H** short-term stability; **I** high cost; **J** long modification of electrode.

## 5. Meta-Analysis on Reviewed Graphene and Its Derivative Sensors and Biosensors

### 5.1. Data and Plotting Pre-Processing and Post-Processing Steps for Method Reproducibility

**Figure 8:**
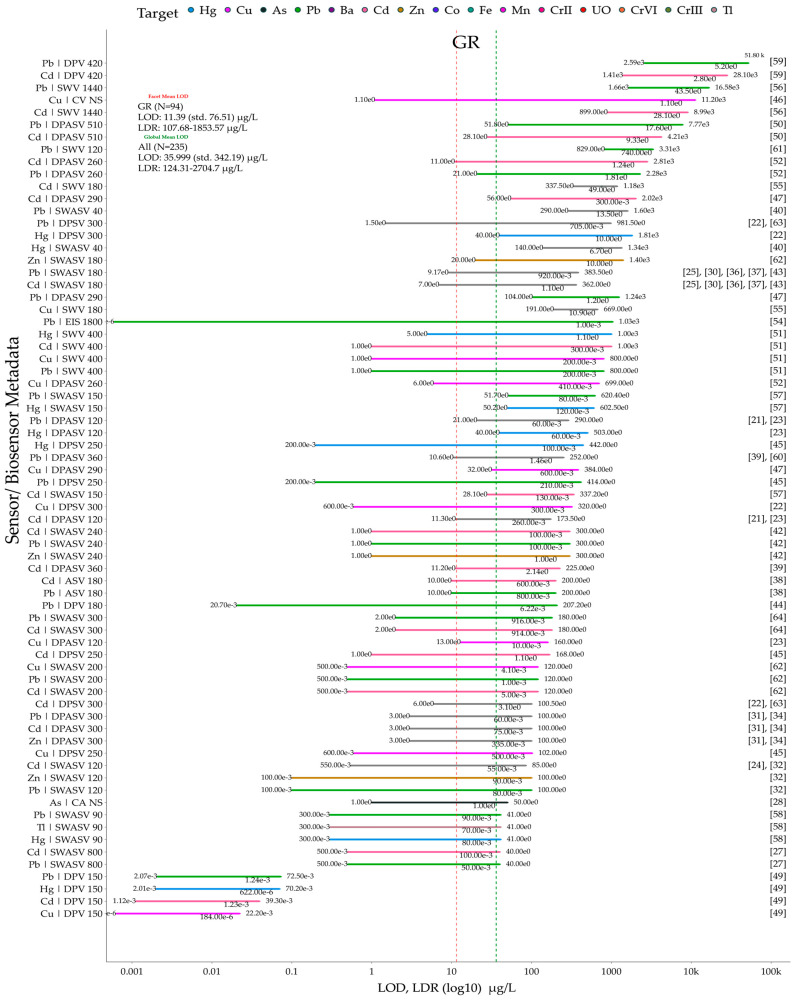
LOD and LDR forest plot of all reviewed graphene-based sensors and biosensors for the electrochemical detection of heavy metals [21,22,23,24,25,27,28,30,31,32,34,36,37,38,39,40,42,43,44,45,46,47,49,50,51,52,54,55,56,57,58,59,60,61,62,63,64].

**Figure 9:**
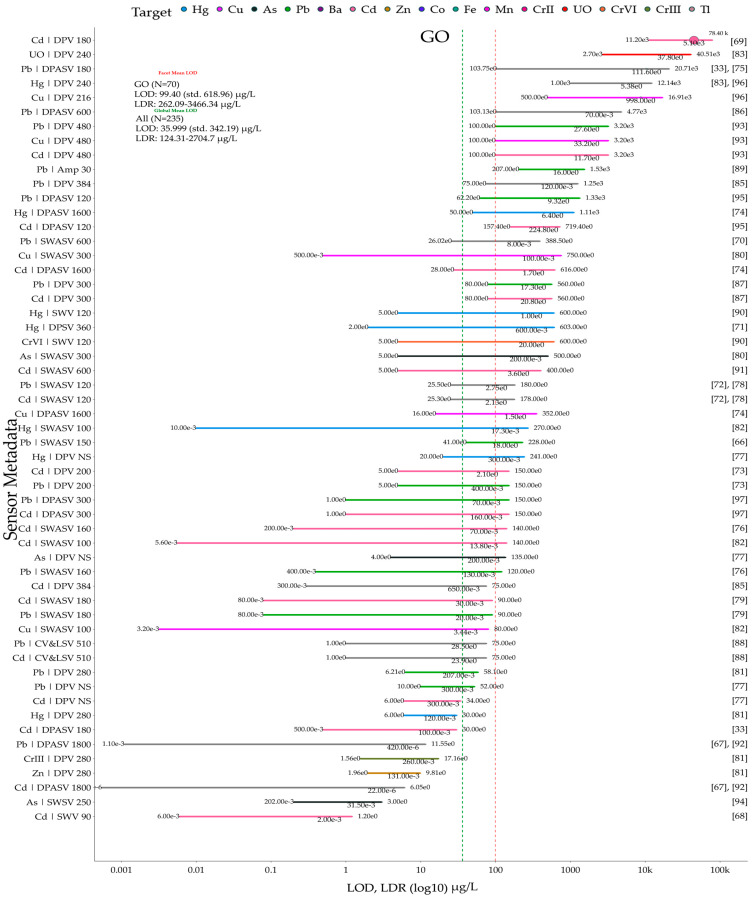
LOD and LDR forest plot of all reviewed graphene oxide-based sensors for the electrochemical detection of heavy metals [33,66,67,68,69,70,71,72,73,74,75,76,77,78,79,80,81,82,83,85,86,87,88,89,90,91,92,93,94,95,96,97].

**Figure 10:**
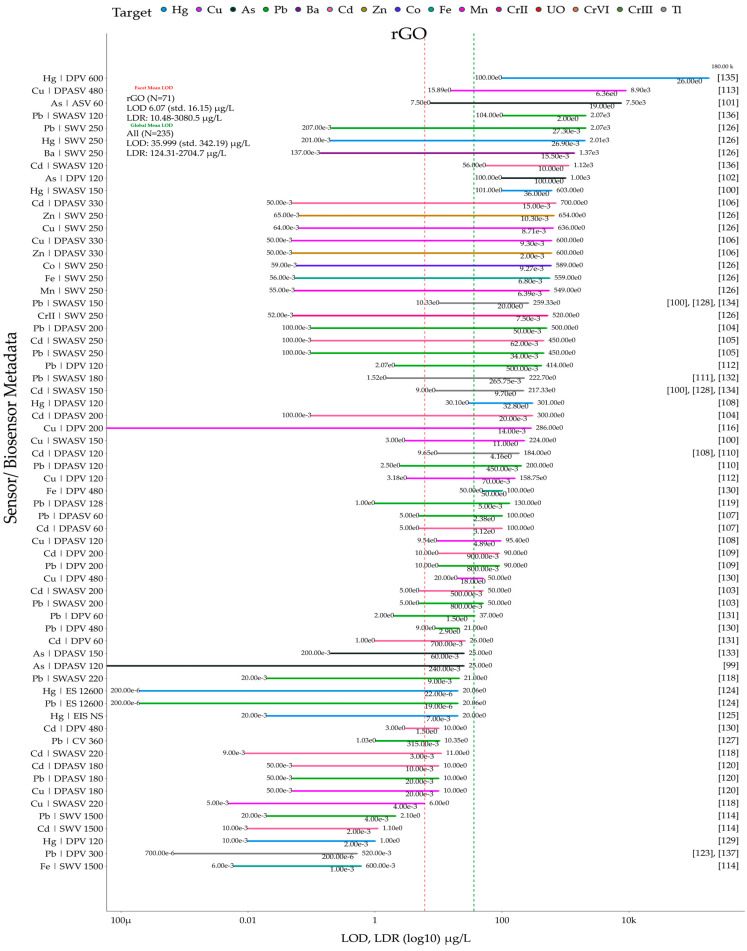
LOD and LDR forest plot of all reviewed reduced graphene oxide-based sensors and biosensors for the electrochemical detection of heavy metals [99,100,101,102,103,104,105,106,107,108,109,110,111,112,113,114,116,118,119,120,123,124,125,126,127,128,129,130,131,132,133,134,135,136,137].

**Figure 11:**
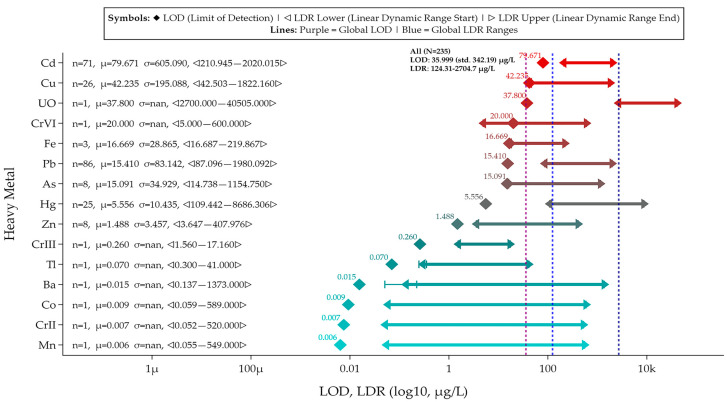
Comparative LOD and LDR performance of all reviewed sensors and biosensors grouped by heavy metal ions.

**Figure 12:**
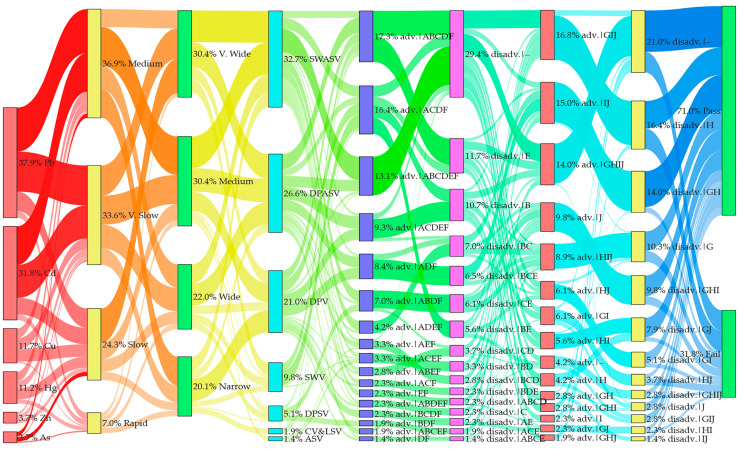
Sankey flow diagram for contributing percentages of categories for all reviewed sensors and biosensors for the electrochemical detection of heavy metals.

A comparison of all reviewed graphene-based sensors and biosensors was performed in the Python programming language (3.9.20). The data was cleaned and organized for processing using NumPy (1.26.4) and Pandas (2.2.3) libraries. Additionally, Figure 8, Figure 9, Figure 10, Figure 11 and Figure 12 were created using Nxviz (0.7.6), Networkx (3.2.1), and Plotly (5.24.1) libraries.

The LOD and LDR values of the sensors were compared for different material types and target heavy metal ions. The pre-processing steps included importing the raw table from Table 1, Table 2 and Table 3 into Excel and exporting as a tab-separated text file and further reimporting the data into Jupyter Notebook (7.2.2) using Pandas. The LDR values were separated into start and end values, and columns were created for LOD, accumulation time, substrate, sensor material, as well as references. For visualization purposes, the start and end points were converted to engineering notation, and the LOD values were log-normalized to be visualized as marker sizes. For categorical classification, the sensors and biosensors were binned using the pandas.cut method. Pre-processing steps included replacing missing values with the median of the column, followed by minimum–maximum scaling between 0 and 1 for the LDR/LOD ratio and natural log normalization for the accumulation time (in seconds). The categorical binning was performed for Rapid (between 0–0.2 quantiles), Medium (between 0.2–0.5 quantiles), Slow (0.5–0.7 quantiles), and Very Slow (<0.7 quantiles). Similarly, the LDR categories binning performed as Narrow (between 0–0.2 quantiles), Medium (between 0.2–0.5 quantiles), Wide (0.5–0.7 quantiles), and Very Wide (<0.7 quantiles). Subsequently, the sensors were ranked for two criteria: (a.) top and bottom based on LDR/LOD ratios alone and (b.) optimum categories across LDR/LOD ratio and detection time.

Furthermore, the coding scheme shown in Table 1, Table 2 and Table 3 was used to create the Sankey flow diagram. The analytical characteristics advantages (adv.) include low LOD (A), wide LDR (B), selective electrode (C), high reproducibility (D), low deposition time (E), and high conductivity (F), while the disadvantages (disadv.) include high LOD (A), narrow LDR (B), non-selective electrode (C), low reproducibility (D), long deposition time (E), and low conductivity (F). Similarly, the sensor characteristics advantages include easy modifier fabrication (G), long-term stability (H), low cost (I), and fast modification of the electrode (J), while the disadvantages include complex modifier fabrication (G), short-term stability (H), high cost (I), and long modification of electrode (J). Firstly, the possible combinations were generated for all unique categories by iteratively traversing the columns of the data frame and then counting the paths for the occurrence of the respective categories. A distinction was maintained between Cr (II), Cr (III), and Cr (VI) as per the International Agency for Research on Cancer (IARC) classification [138], and both Cr (III) and Cr (VI) were annotated according to the WHO LOD Pass/Fail criteria based on the concentrations mentioned in Table 4 and derived from [139].

The Sankey plot was used to analyze the percentage contribution of each entry across all reviewed sensors and biosensors. The data frame consisted of unique paths for each corresponding sensor and biosensor, which represented the inputs for the source and sink values for the Sankey flow diagram. Subsequently, the corresponding values were filtered at count > 5, and vertical positioning was adjusted as the sum of the cumulative flow and individual flow values divided by the total flow through the system. In this way, percentage annotations were generated and displayed.

The post-processing steps were performed to ensure the readability of the meta-analysis figures. Here, the sensors and biosensors were grouped by detection method and time with the corresponding references. Three facets were generated for GR, GO, and rGO sensor and biosensor categories with their corresponding descriptive statistics. For the presentation of the comparative analytical metrics, the sensors and biosensors were grouped according to heavy metal ion categories. All plots were exported as Support Vector Graphics (SVG) with minimal margins and axis labels at scale 2 with A4 paper dimensions using Plotly SVG exporting feature. The global and facet means were computed during plot generation but adjusted in post-processing steps in Adobe Photoshop (24.7.0) to improve readability such that they were displayed in the blank regions of the generated plots rather than over the corresponding lines.

### 5.2. Sensor and Biosensor LOD and LDR Comparison Grouped by Material Types and Heavy Metal Ion Types

The LOD and LDR values (log_10_ scale) were faceted by the material types—graphene (Gr), graphene oxide (GO), and reduced graphene oxide (rGO)—which are shown in Figure 8, Figure 9 and Figure 10. Therefore, the sensors with extremely wide LDRs visually span several orders of magnitude, while those with extremely narrow LDRs appear as small bars. Furthermore, each bar plotted according to the sensors and biosensors metadata also shows the lower LDR at the left end and the upper LDR at the right end, as well as the corresponding LOD in the middle. Values are plotted linearly but positioned along the log-scaled axis. To further improve accessibility, each bar is accompanied by the sensor metadata separated by pipe symbols (|), with entries corresponding to heavy metal and the voltammetric method used with accumulation time in seconds. The unspecified entries for the accumulation time are shown as NS. Finally, the cited reference associated with the corresponding entries on the *y*-axis is shown on the right-hand side of the figures. Entries sharing *y*-axis metadata cite multiple references (right side). For clarity, LOD/LDR values for grouped entries were averaged and displayed in engineering notation. In this regard, the LOD falling within the dynamic range suggests that the given sensor can detect the analyte at the given concentration while also being quantified reliably.

In addition, the faceted figures include two vertical lines, where the green line represents the global average for all reviewed sensors, and the red line represents the grouped average for the material types. If the LOD is below the lower dynamic range, it implies that the detectable concentration is lower than the accuracy of quantification. Finally, an LOD falling above the dynamic range implies that the given sensor may not be sensitive enough to detect the analyte for applicability. The performance of the sensors in each material group was comparable, with rGO sensors performing the best for LODs and the overall dynamic range, as well as the lower end of the dynamic range. Aptasensors performed the best for LOD and LDR, but they were outliers in overall detection time. Therefore, a comparison of the LOD and LDR aids in identifying the suitability of a particular sensor or biosensor for heavy metal ions for the specific environmental applications.

The sensors were also compared for grouping by the metal type, as illustrated in Figure 11. The standard deviation cannot be established for some metal groups due to the small sample size (*n* = 1, shown as nan). The plot shows the LOD and LDR values along a logarithmic *x*-axis. Thicker bars span the lower-to-upper LDR range, with error bars denoting measurement variability. A reference line is shown for the LOD (in purple) and the LDR (in blue). The most sensitive LOD was observed for Mn ions, but note that the sample size is 1. Therefore, caution is advised for generalizations based on singletons. For metals with >10 samples (e.g., Hg), the mean LOD was below the global average, and LDRs were notably wide. Furthermore, the error estimates for many ions, such as Ba and Ti, are not aligned due to the small sample size.

### 5.3. Analysis of the Advantages and Disadvantages According to the Coding Scheme

The Sankey flow diagram shown in Figure 12 demonstrates the overall percentages of each item within their respective categories, with represented paths. All percentage statistics have been annotated programmatically for better readability. The plot is filtered for category count > 5 and sorted on the vertical axis according to largest contribution, where each respective category only displays the overall percentage. For example, 7.0% Rapid implies that 7.0% of entries were binned as Rapid. The most common heavy metals detected were Pb (37.9%), followed by Cd (31.8%) and Cu (11.7%). Furthermore, nearly half (approximately 50%) of the flow originating from the V. Slow category (33.6%) contributes to the V. Wide category. The most common electrochemical method is SWASV (32.7%).

Less than 1% of the reviewed sensors demonstrate all analytical disadvantages. The symbol “–“ serves as a placeholder indicating no explicit advantages. The categories are represented with a pipe symbol (|) with respective advantage or disadvantage for quick inferences. The connections with more advantages will have less flow to disadvantages and vice versa, as these categories are represented by the same letters. A notable analytical disadvantage is narrow LDR (B, 10.7%), and it occurs frequently with ACDF (16.4%). This suggests that sensors exhibiting high LOD, selectivity, reproducibility, and high conductivity are frequently also associated with the narrow LDR category. The most common electrode disadvantages are complex modifier preparation (G) 10.3% and short-term stability (H) 16.4%. Exploring the disadvantages (analytical and sensor/biosensor), G and H (14.0%) represent the percentage of co-occurrence. It can be deduced that the advantages of easy modifier fabrication, low cost, and fast modification of the electrode (GIJ = 16.8%) have an issue of short-term stability (H = 16.4%). Therefore, future sensor fabrication efforts should target the prominent co-occurrence patterns (represented by the thickest flows) identified in the Sankey diagram. Furthermore, the sensors, on average, passed the WHO guidelines for respective heavy meals (71.0%); however, achieving rapid detection with a wide LDR remains a notable challenge.

### 5.4. Identifying Top, Bottom, and Optimum Sensors from the Meta-Analysis

The top, bottom, and optimum performing sensors according to the performed meta-analysis can be identified by accounting for both detection range and time. Considering only the LOD and LDR performance, the top-performing sensors include GO/GCE using DPASV (30 min) in rice, tap water, soya, and milk food products [92], GAs-AuNPs/DNA/ITO using DPV (225 s) in milk [29], AgNPs/GrNPs/GE using SWASV (200 s) in tap water [62], NF@75rGO@ABT/GCE using SWASV (100 s) in cosmetics (nail paint, foundation) and water [82], and PEI/rGO/CS using DPASV (120 s) in river water [119] are the top five performing sensors exhibiting a mix of rapid and slow detection time, predominantly within the very wide range categories. If we expand the list further, MOFs and biosensors (aptasensors) are the top-performing sensors for this group; however, common drawbacks include selectivity challenges, long deposition time, high cost, short-term stability, and long modification of the electrode.

Similarly, the bottom performing sensors include ALA/pDA/rGO/GCE using DPV (480 s) in tap water [130], AC/GP and rGO/CPE using CV and LSV (510 s) in simulated wastewater [88], Co_3_O_4_/GO-2/Nafion/GCE using DPASV (120 s) in lotus pond water [95], GrCFa/GCE using SWV (120 s) in lipstick [61], and NCO/N,S-rGO/GCE using DPASV (120 s) in real water sample [108]. They were the bottom five sensors in terms of LDR/LOD performance. All these sensors were categorized as slow and medium for detection time, narrow LDRs, and non-selectivity, and they shared short-term stability and high cost of manufacturing, while GrCFa/GCE also shared long modification as a flaw.

Finally, the top five optimum sensors (considering both LDR/LOD ratio and detection time) include GR/CeO_2_ DPASV (120 s) in buffer [23], NMO-GR/GCE using SWASV (40 s) in river water [40], PCA-RGO/AuNPs/CSPE using ASV (60 s) in water [101], Bi_2_O_3_/Fe_2_O_3_/GO/PGE using SWV (90 s) in fruits, water, soil, and human serum, IL/GR/L/CPE using SWASV (90 s) in tap and river water and soil [58], and PEI/CNTs/GO/Au using amperometry (30 s) in drinking water [89]. Although the sensors are optimal in terms of range and speed, they have a shared short-term stability issue, with the exception of NMO-GR, which has long-term stability, but the matrix is not complex. GNR-PPy/GCE using DPV (180 s) in lake water [44] and polyP NPs/rGO/CC DPV (120 s) in tap water and lake water [112] are particularly noteworthy for environmental monitoring due to their cost and stability.

Table 1, Table 2 and Table 3 summarize various sensors and biosensors developed for detecting heavy metal ions, covering applications such as water quality assessment, soil contamination analysis, and sediment analysis. These sensors and biosensors have also been researched for food safety in complex test matrices, including cosmetics and food. The practicality of these sensors is limited by the actual LOD and LDR performance in these matrices. In this regard, biosensors such as Aptamer/CS/rGO/TiO_2_ [123] are only suitable for long-term analyses to a limited extent due to low retention. In addition, various trade-offs are observed, for example, GAs-AuNPs [29] have a femtomolar detection time, but the 4 h incubation step is a limiting factor for rapid analyses. Similarly, Bi/Nafion/LIPG [36] is excellent for in situ detection, but it requires UV pre-treatment to remove humic acid, with narrow LDR and a non-uniform calibration for low and high concentrations of the analyte. On a positive note, most of these sensors have qualified WHO pass–fail criteria for heavy metal detection. However, sensors involved in industrial and urban monitoring have a separate bottleneck, particularly long deposition time delays and complex synthesis. Similarly, for remote monitoring, dual-function systems such as N,S-rGO/NiCo_2_O_4_ [108] are useful, but narrow LDR hinders broad applications. Sensors employed in clinical use cases face additional bottlenecks related to analyte stability in the detection medium and pH sensitivity. This usually requires additional sample processing. For example, rGO/AuNPs/SPE [102] has variable recoveries for the detection of arsenic in human serum, and the hydrolysis step reduces stability. An additional complexity is that heavy metal detection, in this case, the ion-imprinted polymers Cys-CTS/N-RGO, is useful [138].

## 6. Toxicity of Heavy Metals

### 6.1. Effect of Toxicity of Heavy Metals on Human

Metals are an essential element of all life forms on Earth, but their contribution to healthy life is on a very fine line. Though metals are required for human biological functions, only a few are essential nutrients. Excessive intake of metal can cause severe health complications. Not only the metals that the human body does not need or that are toxic, but the required metals can also pose a serious risk if the intake is in immeasurable amounts [140]. The essential metals are only required in limited quantities, such as ideal calcium intake is around 700–800 mg per day, potassium is 3.5 mg per day, magnesium 100 mg, sodium 3 g, iron 1–3 mg, zinc 15 mg, copper no more than few mg in a day, manganese 1–10 mg, molybdenum 0.3 mg, cobalt few μg in a day, and tin 0.3 mg [141]. Humans are mainly exposed to these toxic metals through food. These heavy metals contaminate food during the manufacturing or packaging process or by coming from a contaminated food source [142]. Metal-contaminated water and insecticides or fertilizers are the major sources of food contamination.

#### 6.1.1. Cadmium’s Impact on Human Health

Cadmium is a toxic heavy metal with carcinogenic effects, and the biological half-life of cadmium in the human body ranges, on average, from 16 to 30 years [140]. The main pathway of cadmium exposure for humans is the respiratory pathway, but it can also be absorbed from the digestive system through contaminated food. As per estimations, 13–19% of cadmium is absorbed in the lungs, and 10–44% is absorbed through the intestine, mainly within the small intestine. Exposure to higher levels of cadmium toxicity during pregnancy leads to developmental problems in the foetus and placenta. Cadmium can cross the placental barrier, which leads to intergenerational and multigenerational epigenetic changes and can also lead to embryonic death. Cadmium has been observed as a regulator of DNA methylation and bone mineralization; therefore, the effects of this HM can last several generations [143].

#### 6.1.2. Chromium’s Impact on Human Health

Chromium is a carcinogenic heavy metal that adversely affects health, depending on the exposure routes. The human body can be exposed to chromium by ingesting contaminated food or water, inhaling chromium particles from the air, and by direct skin contact. Chromium has been linked to replication stress-associated DNA damage. Chromium inhalation leads to lung problems such as tumours, pneumonia, pulmonary fibrosis, lung neoplasm, and lung cancer [144]. Short-term chromium exposure causes diarrhea, nausea, vomiting, pupil dilation, and lacrimation. Chromium toxicity is associated with respiratory, gastrointestinal, renal, and immune system disorders. Fatalities due to chromium toxicity have also been reported [145].

#### 6.1.3. Mercury’s Impact on Human Health

Mercury is one of the most mentioned toxic heavy metals, as it has widespread long-term and fatal effects on human health. The main source of mercury intake in the human body is marine and freshwater foods; however, the recent literature also points to other dietary sources [146]. Mercury induces transgenerational epigenetic changes, impairing neurocognitive development and increasing susceptibility to neurocognitive disorders [147]. Seafood is one of the major sources of methylmercury in food. Accumulation of mercury is present in small to large species of marine life, but the larger predatory species have higher concentrations of mercury in their bodies, and consumption of these fish or seafood leads to the accumulation of mercury in the human body. Swordfish, shark, king mackerel, bluefin tuna, and tilefish have the highest concentration of mercury. Among spices and herbs, cloves and mustard possess the highest concentrations of mercury [148].

#### 6.1.4. Lead’s Impact on Human Health

Lead is a non-biodegradable and persistent heavy metal with toxic effects on humans, animals, plants, and even microorganisms. Lead is responsible for damage to the cardiovascular system, renal and gastrointestinal tract, and nervous system in humans [149]. The exposure of children to lead has negative effects on their development. In particular, the neuropsychological development of children is affected by long-term lead exposure, leading to behavioural abnormalities and declined IQ levels [150]. High levels of lead toxicity result in anemia, excessive weight loss, lethargy, seizures, coma, and ultimately, death [151].

#### 6.1.5. Zinc’s Impact on Human Health

Zinc is a necessary metal for the human body that serves several important roles. Zinc is involved in immunity mechanisms, reproduction, muscle development, intestinal function, glycolipid metabolism, and even nervous system function and development. Zinc is also a key component of the DNA repair mechanism. However, excessive intake of zinc leads to zinc toxicity, which disrupts the natural regulation of zinc metabolism in the human body and leads to neurodegenerative disorders, immune system dysfunction, reproductive disorders, and even cancer [152]. An imbalance of zinc in the human body leads to oxidative stress, cellular degeneration, and abnormal apoptosis. Zinc dysregulation and toxicity are associated with several neurodegenerative diseases, such as Alzheimer’s, Parkinson’s, Huntington’s, epilepsy, and stroke [153]. Zinc acts as a cofactor for the enzymes involved in DNA expression, membrane stabilization, vitamin A metabolism, and gustatory and olfactory systems [154].

#### 6.1.6. Arsenic’s Impact on Human Health

Arsenic is a metalloid that occurs in the Earth’s crust and is released into the environment through geological, industrial, and agricultural processes. Arsenic is highly toxic to humans. Prolonged exposure to arsenic has serious effects on the cardiovascular, nervous, endocrine, respiratory, reproductive, and haematological systems. Human health problems associated with chronic exposure to arsenic are known as “arsenicosis’’. Bell-Ville disease, Blackfoot disease, and Kai Dam disease are also associated with chronic arsenic toxicity [155]. Arsenic generates intracellular reactive oxygen species (ROS), which are responsible for altering cell signalling pathways, oxidative stress, and epigenetic mutations, leading to DNA damage, cellular dysfunction, hormonal changes, and cancer [156]. Arsenic-induced mutagenic oxidative DNA damage leads to various types of cancer. The IARC has classified arsenic as a Group 1 carcinogen. Several reports follow the WHO and the Agency for Toxic Substances and Disease Registry (ATSDR), which have provided evidence for the organ-specific carcinogenicity of arsenic and its contribution to several types of cancer [157].

### 6.2. Effects of Toxicity of Heavy Metals on Plants

Heavy metals are an important factor for toxic symptoms in plants. Heavy metal toxicity impairs plant growth, causing chlorosis, reduced biomass, photosynthetic deficits, and death [158]. Heavy metals cause oxidative stress in plants by producing ROS. The toxicity of Pb and Cu in plants leads to an increase in ROS levels, and further, a decrease in the photosynthetic efficiency due to membrane damage and lipid peroxidation. Moreover, As and Hg affect cell metabolism in plants by altering protein and enzyme activities. HMIs such as Cd, Cr, Cu, Pb, and Hg cause malnutrition and death of plants [159]. Heavy metals also disrupt biotic pollination by reducing nectar quality and pollinator attraction, impairing plant reproductive fitness. The altered chemical properties of nectar cause pollinators to avoid and negatively impact the flower life cycle, resulting in deterioration of the plant–animal ecosystem [160]. Heavy metals also accumulate in the plant body, which later circulates in the ecosystem and has persistent toxic effects.

### 6.3. Effects of Toxicity of Heavy Metals on Soil

Contamination of the soil with heavy metals is a major problem for the environment. Heavy metals disrupt soil chemistry and microbial activity, causing bioaccumulation, ecosystem toxicity, and reduced plant growth. The microorganisms in the soil play a crucial role in the recycling of substances and in maintaining the energy flow of the ecosystem, as these microorganisms are highly sensitive to heavy metals. Contamination of the soil with heavy metals leads to a decline in soil microbiome, which negatively impacts plant growth and the sustainability of the ecosystem [161]. Cd, Pb, and As reduce microbial biomass by altering microbial metabolic activity and enzyme functions [159]. Heavy metals alter soil chemistry by changing the soil pH, nutrient balance, and redox reactions and reducing the adsorption properties of liquids in the soil [162]. These heavy metals from the soil enter the human body via plants, water, and other food sources. Soil sequesters environmental heavy metals, which later leach into groundwater. Heavy metals in the soil enter the groundwater by diffusion [163] and contaminate the air through soil erosion, which affects the entire ecosystem.

### 6.4. Effects of Toxicity of Heavy Metals in Aquatic Life

Anthropogenic activities are the main source of heavy metals in water and disrupt the natural aquatic ecosystem. Heavy metals bioaccumulate in aquatic organisms, ultimately contaminating humans through the food chain. HMs such as Pb, Hg, Cu, and Zn have a detrimental effect on the growth and reproduction of fish and alter their feeding and mating behaviour [164]. Larval and embryonic stages of fish are affected by heavy metal exposure, leading to spinal deformities, reduced cardiac function, and high mortality rates [165]. A 2020 study by Islam et al. [166] reported detrimental effects of Cr in fish, where Cr was responsible for altering the haemato-biochemical composition, and deformations were observed in the nuclear and cellular structure of blood cells [166]. Heavy metals disrupt shell mineralization in molluscs (e.g., scallops, mussels, oysters), keystone species in marine ecosystems. Zn, Cu, and Pb are responsible for the deterioration of shell strength of molluscs, leading to their mortality and affecting the natural food chain and ecosystem [167]. Aquatic plants also accumulate heavy metals and, through the food chain, transfer them to other organisms. Heavy metal accumulation in aquatic plants serves as a contamination marker as well [168].

## 7. Contribution of Heavy Metals to the SDGs

The SDGs [169] are 17 goals that were adopted by the United Nations in 2015 and are to be achieved by 2030 for global development, peace, and prosperity. These SDGs are aimed at solving global problems such as hunger, poverty, health, pollution, clean energy, and climate change. Heavy metals and heavy metal toxicity are implicitly or explicitly linked to some of these goals.

### 7.1. Contribution of Heavy Metals in SDG1 (No Poverty)

Heavy metal poisoning and poverty are interlinked. The effects and accumulation of heavy metals are particularly evident in poor and marginalized communities. Waste dumping sites and industrial facilities are often located near low-income neighbourhoods. The lack of clean water, contaminated crops, and food are factors that primarily affect low- and middle-income demographics [170]. Poverty hinders access to medical care, clean water, and food, as well as education about self-sufficiency and the dangers of toxins. These effects extend beyond individual health to undermine socioeconomic stability. The impact of heavy metal contamination leads to an increase in healthcare costs and a decline in productivity due to illness [171]. Crocetto et al. [172] discussed the relationship between heavy metals and male fertility and their negative impact on long-term socioeconomic development. Heavy metal toxicity impairs male fertility, leading to a demographic decline, disrupted generational turnover, and potentially serious economic consequences. A study by Geron et al. [173] shows a higher toxic effect of heavy metals in women from low-income, higher poverty, and lower education demographics. These factors show a vicious cycle of HM toxicity associated with poverty. The role of heavy metals in socioeconomic development needs to be understood and effectively mitigated in order to achieve the United Nations’ goal of no poverty.

### 7.2. Contribution of Heavy Metals in SDG2 (Zero Hunger)

The effects of heavy metals on plants and animals have been previously discussed. The toxicity of heavy metals negatively affects plant growth and crop production. Animals and aquatic organisms are also negatively affected by heavy metal toxicity, which damages the food chain. Heavy metals are absorbed by plants from contaminated soil and water [174]. These metals accumulate in plant tissue, especially in roots, stems, and leaves, and thus enter the human food chain [175]. Heavy metals degrade nutritional quality and cause toxicity in consumers [176]. For sustainable management of world hunger, it is important to reduce heavy metal toxicity in order to improve human food sources and minimize the risk of HM contamination from food.

### 7.3. Contribution of Heavy Metals in SDG3 (Good Health and Well-Being)

Heavy metals pose a major health risk, previously discussed in detail. The effects of heavy metals on health and well-being can be assessed by their bioaccumulation and toxic output. Heavy metals are toxic to all living organisms. Their threshold level may vary, but overall, they pose a health hazard. Some of the HMs, such as As, Cd, Hg, Pb, Ni, and Cr, are known to be teratogenic and have been categorized as carcinogens. As, Cd, Cr, and Ni have been classified as group 1 carcinogens (Table 4) by the WHO, ATSDR, and IARC. These toxic heavy metals generate ROS that promote oxidative stress, genotoxicity, mutagenic changes, and DNA methylation, leading to various types of serious health problems [177]. Heavy metal toxicity affects adults, children, and even foetuses through various pathways, leading to physical and mental issues [178,179]. For sustainable development in good health and well-being, the consideration of heavy metal toxicity as a key factor is necessary.

### 7.4. Contribution of Heavy Metals in SDG6 (Clean Water and Sanitation)

Heavy metals contribute significantly to the contamination of groundwater and surface water. Water is contaminated by the deposition or leaching of heavy metals from the soil [180]. Bioaccumulation and absorption are the most important transport mechanisms for HMs in water [181]. Sanitation is another factor that affects heavy metal contamination in water. Traditional sanitization methods are not effective in removing heavy metals from water. Conventional methods of wastewater treatment are often ineffective against heavy metals due to the small size and persistence of the particles [182]. Given the far-reaching lethality of heavy metal contamination, securing clean water is urgent. To fulfil the SDG6 goal, we need to prioritize the mitigation of HM contamination and improve remediation. This is a key factor that can jeopardize sustainability efforts.

### 7.5. Contribution of Heavy Metals in SDG7 (Affordable and Clean Energy)

One of the main causes of anthropogenic heavy metal pollution is mining. Power generation facilities such as thermal power plants and nuclear power plants depend on raw materials extracted from mining, releasing HMs into the environment. Hydropower generation is also linked to HM pollution. Dams serve not only as water reservoirs but also as sinks for heavy metals and metalloids, which are deposited in high concentration in the sediments and are also highly diluted in the overlying water [183]. These heavy metals then affect the natural ecosystem of the dam and the surrounding and distant areas by flowing out of the dam. In addition, sedimented heavy metals leach into the soil at high levels and contaminate the groundwater reserves, where the pressure of the dam facilitates absorption. Clean energy alternatives such as solar energy, geothermal energy, and wind energy, among others, are not free from heavy metals. The main sources are batteries and common electrical equipment, such as cables that contain high levels of heavy metals. Batteries contain Pb, Cr, Cd, and Ni, and electrical cables contain Cu, Zn, and Cr. The improper disposal of these leads to heavy metal pollution of the environment [184]. Affordable and clean energy for everyone is a prerequisite for sustainable development, and the control and mitigation of environmental toxic hazards from these energy sources, especially heavy metals, are of paramount importance. There is an urgency for proper management and legislation for long-term sustainability.

### 7.6. Contribution of Heavy Metals in SDG9 (Industry, Innovation, and Infrastructure)

Modern infrastructure and innovation are the building blocks of future development, and industry is the key component for sustainability. The future of mankind and nature is dependent on innovation, green infrastructure, and rapid development of industries, as all aspects of human life are interconnected with these three prospects. Technical advancements and innovations are crucial for maintaining efficiency and reducing environmental effects. In this context, industries are the manufacturing hubs to utilize the positive effects of new innovations and their distribution among people. Infrastructures like transportation, energy, and healthcare are essential to maintain innovation and industry growth. The UN reports have highlighted post-pandemic growth in industry, infrastructure, and innovation for a more sustainable future. While these areas are essential for long-term SDGs, they are also the critical points for heavy metal contamination. Industry, innovation, and infrastructure rely on non-biodegradable metals. These accumulate in biomass, soil, and water, causing long-term harm [185]. The immediate restrictions on usage of HMs in these sectors are not possible; however, a need to look for new technologies for mitigating HMs and their environmental impacts should be prioritized.

### 7.7. Contribution of Heavy Metals in SDG11 (Sustainable Cities and Communities)

One of the goals of the UN is to make cities, communities, and settlements safe and sustainable. To achieve this goal, extensive measures need to be taken for municipal and urban development, pollution control, food, water, medical facilities, and other infrastructure. The biggest problem in most cities today is pollution, and heavy metals are among the pollutants responsible. For this reason, heavy metals are directly linked to the goal of “sustainable cities and communities”. The elimination of heavy metals, the minimization of impacts, and the elimination of soil, air, and water pollution are necessary for the development of sustainable cities and communities [186].

### 7.8. Contribution of Heavy Metals in SDG12 (Responsible Consumption and Production)

Responsible consumption and production are one of the objectives for sustainable development in relation to heavy metals. As mentioned above, the main anthropogenic sources of heavy metals are mining, industry, power plants, infrastructure development, and the use of chemicals in agriculture. These sources are controlled by the need for human consumption. Meanwhile, expenditures on containment, remediation, and healthcare undermine the sustainability of the sustainable development of societies. By controlling electricity consumption, utility usage, and the production and use of chemicals, we can control the societal expenditures, redirecting them towards sustainability and also curbing HM pollution.

### 7.9. Contribution of Heavy Metals in SDG13 (Climate Action)

Heavy metals are directly linked to climate change. Soil heavy metal contamination disrupts microbial activity, slowing organic matter decomposition, which releases greenhouse gases like CH_4_ [187]. Heavy metals affect chlorophyll synthesis and the natural development of plants, leading to a CO_2_ imbalance. In addition, heavy metal toxicity alters the pH of water in rivers and coastal areas, leading to the disruption of photosynthesis and CO_2_ absorption efficiency, which, in turn, leads to the acidification of seawater [188]. For these reasons, the United Nations SDGs for climate change mitigation should specifically consider the risks and mitigation strategies associated with heavy metals.

### 7.10. Contribution of Heavy Metals in SDG14 (Life Below Water)

SDG14 is critical for long-term sustainability. Not only does aquatic life have a huge impact on human life as a food source, but it also contributes to ecological balance and environmental sustainability. Heavy metal toxicity has a rapidly growing negative impact on marine life. Heavy metals disrupt marine ecosystems and have a physiological impact on marine flora and fauna [189]. Marine ecosystems contaminated with HMs lead to ecological imbalance, global warming, and risks to human health [190]. Heavy metals contribute directly to some of the targets of SDG14, in particular, reducing marine pollution, reducing ocean acidification, conserving coastal and marine areas, and protecting and restoring ecosystems.

### 7.11. Contribution of Heavy Metals in SDG15 (Life on Land)

Life on land is another goal of the SDGs: protect and restore ecosystems, sustainably manage forests, combat forest desertification, and halt biodiversity loss. Heavy metals and metalloids have a strong impact on the land ecosystem by polluting the soil and negatively affecting flora and fauna [191]. The disruption of soil microorganisms by heavy metal contamination leads to ecological damage [161]. Heavy metals are also responsible for the impairment of plant growth, animal health, and the loss of biodiversity. Heavy metals are toxic to plants, animals, and humans and have a negative impact on the entire ecosystem [192]. For the sustainable development of life on land and the ecosystem, there is an urgent need to mitigate the effects of heavy metal contamination.

### 7.12. Contribution of Heavy Metals in SDG17 (Partnerships for the Goals)

International cooperation among countries in research and development for the sustainable management of heavy metals can promote global sustainable development. Countries ought to work together to achieve the sustainable goals by eliminating the impact of heavy metals in the ecosystem, their release in the environment, and their toxicity through innovative solutions, intervention, and detection technologies. Innovations like advanced sensors will enable rapid heavy metal detection and toxicity reduction of heavy metals in the medical field.

## 8. Summary and Outlook

This comprehensive review has critically examined the advances in electrochemical sensors based on graphene and its derivatives for the detection of heavy metals in real-world products, highlighting their transformative potential in addressing global environmental challenges. Electrochemical sensors based on graphene and its derivatives are more sensitive in terms of rapid detection of heavy metals due to their large surface area and conductivity. Among the voltammetric methods, SWASV was the most commonly used electrochemical method. In addition, aptasensors and metal–organic frameworks showed the highest sensitivity among the analysed sensors. However, complex modifier fabrication requiring multi-step processes to enhance conductivity remains a common drawback. Complex modifiers and costly nanoparticles hinder scalability and raise manufacturing expenses. Despite the significant improvement in selectivity in the determination of heavy metals with graphene-based sensors, numerous development tasks still need to be addressed.

Electrochemical sensors require nanoscale optimization for cost-effective, high accuracy. In addition, most current research work is limited to analyzing samples in the laboratory. Sensor-based detection of heavy metals should fulfil the conditions for on-site monitoring. Further research should focus on the development of sensors that can be integrated into mobile devices to enable rapid on-site detection. The additional integration of edge computing techniques, cloud platform solutions, and the support of machine learning algorithms can increase the performance of the sensors and biosensors. In this context, systems based on artificial intelligence and machine learning algorithms need to be implemented for rapid analysis of complex multimodal sensor data. However, there are still some problems with inorganic nanomaterials, which are relatively expensive and difficult to mass produce, while some organic materials are less stable and toxic. In addition, the use of biomaterials is limited to optimal conditions. The high surface conductivity of graphene sensors, which amplifies the electrochemical signal of the analysis, is still limited in terms of reproducibility and selectivity. This can be achieved through advances in manufacturing techniques such as laser engraving.

The analyzed sensors based on graphene derivatives met the WHO guidelines on average. rGO sensors outperformed others in LOD/LDR, though material groups showed comparable overall performance. Additionally, most studies focus on the detection of Pb, Cd, and Cu. In contrast, the remaining ions account for less than 15% of all sensors analyzed, highlighting the discrepancy in the heavy metal ions targeted. Furthermore, the multiplexity of sensors is being developed in many studies, with a major challenge being the elimination of intermetallic interference. With appropriate categorical coding, we can also quantitatively discuss the advantages and disadvantages of a path-based approach. The proposed path-based Sankey flow diagram is a quick reference tool to highlight the important trends, and the logarithmic LOD-LDR comparison forest plots provide quick insights into the sensor performance.

The meta-analysis provides various insights for sensor and biosensor use cases in environmental monitoring. The critiques analyzed correspond to the three barriers to the adoption of sensors and biosensors for monitoring purposes, including stability, cost, and selectivity. The detection step is the final bottleneck for real-time detection, regardless of the portability of the device. In this context, the choice of substrate is crucial for sensor portability. In addition, sensors and biosensors with validated matrix effects can be used for food safety purposes, where portability is not an issue. Due to interferences and cross-reactions between biological and chemical molecules in some samples (blood, urine), the detection of voltammetric signals becomes complicated. To overcome this problem, future research should focus on the development of advanced electrode materials to increase selectivity and sensitivity. The sensor surface should be functionalized with biomolecules to increase sensitivity and modifiability. Automation through microfluidic integration is also useful for in situ sensor applications. However, sensors for environmental monitoring require stability for long-term performance. In this regard, further advances in the modification of sensor materials are needed to achieve scalability for mass production of sensors.

Another important aspect is the reporting standard for developed electrochemical sensors and biosensors. Authors should report on sensitivity, reproducibility, repeatability, and recoveries. Apart from the above challenges, some issues related to the detection itself, including inconsistent calibration, should also be addressed. The variable performance of the sensor at low and high concentrations leads to performance issues with LDR (related to the detection object). Finally, the choice of nanomaterial also has an impact on detection sensitivity. This is also related to the cost bottleneck. The functionalization and modification of the sensor surface to increase conductivity remains an active area of research.

This review has highlighted the toxic effects of heavy metals on flora and fauna, ecotoxicity and the chain of contamination leading to ecosystem degradation on land and in the sea, the impact on global warming, and the technical aspects related to the detection, analysis, and monitoring of heavy metals. We emphasized heavy metals due to their environmental and human health risks. Responsible consumption can help to mitigate the anthropogenic factors of heavy metal pollution in the environment.

## Figures and Tables

**Table 4:** WHO drinking water safety thresholds and the respective IARC carcinogen group for prevalent heavy metals.

Heavy Metal	WHO Thresholds (Drinking Water) (μg·L^−1^)	IARC Carcinogen Classification
Cd	3	Group 1
Pb	10	Group 2B
Cu	2000	Group 3
Hg	6 (inorganic)	Group 3
Tl	0.1	Group 3
As	10	Group 1
Cr^3+^ and Cr^6+^	50 (total)	Group 1 (Cr^6+^)
Ba	700	Group 3

## Abbreviations

The following abbreviations are used in this work:

1D	One-dimensional
2D	Two-dimensional
3D	Three-dimensional
3DGO	Three-dimensional graphene oxide
AA	Ascorbic acid
ABT	2-(Anthracen-9-yl)benzothiazole
Ag/GDs/NCs/CFs	Silver nanoclusters, graphene quantum dots, N-doped carbon
AgNPs	Silver nanoparticles
ASV	Anodic stripping voltammetry
AuNFs	Gold nanoflowers
AuNPs	Gold nanoparticles
B/N	Boron/Nitrogen co-doped
Bi	Bismuth
Bi NPs	Bismuth nanoparticles
C_3_N_4_	Carbon nitride
CA	Chronoamperometry
CAR	Calixarene
CBT	Benzothiazole-2-carboxaldehyde
CC	Carbon cloth
CFs	Carbon fibers
CHA	Catalytic hairpin self-assembly
CMO	Copper manganate
Co_3_O_4_	Cobalt oxide
COFs	Covalent organic frameworks
COF-SH	Hydrosulphonyl-functionalized covalent organic framework
CS	Chitosan
CS/PANi–Bi	Chitosan/polyaniline–bismuth nanoparticle
DPASV	Differential pulse anodic stripping voltammetry
DPV	Differential pulse voltammetry
EBFC	Enzyme biofuel cell
EDTA	Ethylenediaminetetraacetic acid
EIS	Electrochemical impedance spectroscopy
ErGO	Electrochemically reduced graphene oxide
EUON	European Union Observatory for Nanomaterials
Exo	Exouclease
FDA	United States Food and Drug Administration
Fe_3_O_4_	Iron oxide (magnetite)
f-GO	Functionalized graphene oxide
GAs	Graphene aerogel
GCE	Glassy carbon electrode
GDs/GQDs	Graphene quantum dots
GDY	Graphdiyne
GE	Graphite electrode
GF	Graphene foam
GF/GNFs	Nanoflower-decorated microporous graphene foam
GNFs	Graphene nanoflowers
GNR	Graphene nanoribbon
GO	Graphene oxide
GOMIP	Styrene/EGDMA polymer + functionalized GO
GP	Graphene paper
GR	Graphene
GrCF	Graphene chitosan and Fe composite
GrNPs	Graphene nanoplates
HMs/HMIs	Heavy metals/heavy metal ions
HRP	Horseradish peroxidase
HPLC	High-performance liquid chromatography
ICP	Inductively coupled plasma
IDE	Interdigital electrode
IIPs	Ion-imprinted polymers
IL	Ionic liquid
IL-rGO	Ionic liquid–reduced graphene oxide
ITO	Indium tin oxide
L-cys	L-cysteine
LDR	Linear dynamic range
LIG	Laser-induced graphene
LIGBN	Bismuth and nitrogen co-doped laser-induced graphene
LIPG	Laser-induced porous graphene
LOD	Limit of detection
LRGO	Laser-reduced graphene oxide
MGCE	Magnetic glassy carbon electrode
MGO-PANI	Polyaniline/graphene oxide/polyethylene glycol/cysteine
MnO_2_	Manganese dioxide
MOFs	Metal–organic frameworks
MoS_2_	Molybdenum disulfide
MWCNTs	Multi-walled carbon nanotubes
N,S-rGO	Nitrogen/sulfur co-doped reduced graphene oxide
NF@rGO	Nickel ferrite/reduced graphene oxide
NGA	Nitrogen-doped graphene aerogel
N-GR	Nitrogen-doped GR
NH_2_-MOF	Amino-functionalized metal–organic framework
NiCo_2_O_4_	Nickel cobaltite
NMO	Nickel manganate
NPC	Nanoporous carbon
NPs	Nanoparticles
N-rGO	Nitrogen-doped reduced graphene oxide
NS	Not specified
PAMAM	Poly(amidoamine)
PANI	Polyaniline
PCA	1-pyrene carboxylic acid
PEI	Polyethylenimine
PEI-rGO	Polyethylenimine-reduced graphene oxide
PGA	Poly(L-glutamic acid)
PGE	Pencil graphite electrode
PLL	Poly-L-lysine
PMDA	Poly methyldopa
PolyP	NPs
PPy	Polypyrrole
P-rGO	Phosphorus-doped reduced graphene oxide
PtNPs	Platinum nanoparticles
rGO/RGO	Reduced graphene oxide
ROSES	RepOrting standards for Systematic Evidence Syntheses
RSD	Relative standard deviation
Sb_2_WO_6_	Antimony tungstate
SPCE	Screen-printed carbon electrode
SPE	Screen-printed electrodes
ssDNA	Single-stranded DNA
STB/Gs	Sulfur-doped carbon nitride tube bundles/graphene nanosheets
SWASV	Square wave anodic stripping voltammetry
SWV	Square wave voltammetry
TEOA	Triethanolamine-functionalized
T-GO-C	Thymine/carbohydrazide-functionalized graphene oxide
THI	Thionine
TiO_2_	Titanium dioxide
TPPS	Tetraphenylporphyrin sulfonate
UNEP	United Nations Environment Program
UN	United Nations
WHO	World Health Organization
ZIF-67	Zeolitic imidazolate framework-67
ZIF-8	Zeolitic imidazolate framework-8
ZMO	Zinc manganate
ZnO	Zinc oxide
ZnSe	Zinc selenide
